# Life-Course Psychosocial Stress and Risk of Dementia and Stroke in Middle-Aged and Older Adults

**DOI:** 10.1001/jamanetworkopen.2025.56012

**Published:** 2026-01-28

**Authors:** Bowen Chen, Erxu Xue, Yining Li, Enze Tang, Yaojie Wang, Yue Wu, Shan Liu, Jianhui Zhao

**Affiliations:** 1 The Fourth School of Clinical Medicine, Zhejiang Chinese Medical University, Hangzhou, Zhejiang, China; 2 Center of Clinical Evaluation, The First Affiliated Hospital of Zhejiang Chinese Medical University, Zhejiang Provincial Hospital of Chinese Medicine, Hangzhou, Zhejiang, China; 3 School of Public Health, Zhejiang Chinese Medical University, Hangzhou, Zhejiang Province, China; 4 The D. H. Chen School of Universal Health, Sir Run Run Shaw Hospital, Zhejiang University School of Medicine, Hangzhou, Zhejiang, China; 5 Department of Radiology, The Second Xiangya Hospital, Central South University, Changsha, China; 6 Department of Psychology and Behavioral Sciences, Zhejiang University, Hangzhou, China; 7 Department of Psychiatry, Brigham and Women’s Hospital, Harvard Medical School, Boston, Massachusetts; 8 Department of Preventive Medicine, Northwestern University Feinberg School of Medicine, Chicago, Illinois; 9 Department of Epidemiology and Health Statistics, School of Public Health, Zhejiang University School of Medicine, Hangzhou, China; 10 Key Laboratory of Integrated Care for Geriatric Chronic Diseases, School of Nursing, Kunming Medical University, Kunming, China; 11 Clinical and Translational Epidemiology Unit, Massachusetts General Hospital, Harvard Medical School, Boston, Massachusetts

## Abstract

**Importance:**

The associations of adverse childhood experiences (ACEs) and adverse adulthood experiences (AAEs) with incident dementia and stroke in the Chinese population are not well understood.

**Objectives:**

To investigate the associations of ACEs and AAEs with dementia and stroke incidence, and to examine whether depression mediates these associations.

**Design, Setting, and Participants:**

This population-based cohort study utilized data from the China Health and Retirement Longitudinal Study (June 2015 to December 2020). Participants aged 45 years and older with complete adverse experience data were included and were followed up for a mean (SD) of 4.89 (0.48) years for dementia and 4.84 (0.57) years for stroke. Statistical analysis was performed from August 20, 2025, to November 23, 2025.

**Exposures:**

ACEs and AAEs were assessed through a structured questionnaire, with cumulative scores calculated for both categories.

**Main Outcomes and Measures:**

Dementia was identified using a standardized cognitive battery and activities of daily living scale, while stroke was determined through self-reported physician diagnosis. Depression was evaluated using the 10-item Centre for Epidemiologic Studies Depression Scale. Cox proportional hazards regression analysis was used to explore the association of ACEs and AAEs with the risk of new-onset dementia and stroke, with results presented as hazard ratios (HRs) with 95% CIs.

**Results:**

Among 11 601 participants (mean [SD] age, 59.18 [9.41] years; 5569 male [48.0%]), 9145 (78.8%) were exposed to at least 1 ACE indicator, 4241 (36.6%) to at least 1 AAE indicator, and 3531 (30.4%) to both ACE and AAE markers. Both ACEs (HR, 1.11; 95% CI, 1.05-1.18) and AAEs (HR, 1.23; 95% CI, 1.14-1.33) were significantly associated with higher hazards of dementia during follow-up, whereas only AAEs were associated with higher hazards of stroke (HR, 1.19; 95% CI, 1.12-1.26). Latent class analysis identified a high-risk ACEs subgroup associated with incident stroke (HR, 1.33; 95% CI, 1.08-1.65). In the joint effects analyses, participants in the high-risk groups for both ACEs and AAEs exhibited higher hazards of dementia (HR, 3.28; 95% CI, 1.54-7.02) and stroke (HR, 2.50; 95% CI, 1.24-5.30). Depression mediated 34.3% of the association of ACEs with dementia (β = 0.10; 95% CI, 0.04-0.17), 20.9% of the association of AAEs with dementia (β = 0.22; 95% CI, 0.13-0.30), and 17.5% of the association of AAEs with stroke (β = 0.18; 95% CI, 0.11-0.24).

**Conclusions and Relevance:**

In this cohort study, exposure to adverse experiences throughout life was associated with increased risks of dementia and stroke, with depression mediating these associations. These findings highlight the importance of implementing life-course interventions that address both psychological trauma and mental health to reduce the burden of neurovascular diseases.

## Key Points

**Question:**

Are adverse childhood experiences (ACEs) and adverse adulthood experiences (AAEs) associated with an increased risk of incident dementia and stroke in middle-aged and older adults in China?

**Findings:**

In this cohort study of 11 601 adults, exposure to high levels of ACEs and AAEs was significantly associated with increased dementia risk, with AAEs also being associated with increased stroke risk; depression partially mediated these associations.

**Meaning:**

These findings suggest that life-course adverse experiences may increase the risks of dementia and stroke partly through depression, underscoring the importance of early identification of psychosocial stressors and preventative strategies aimed at mental health and trauma support.

## Teaser

This cohort study examines associations of adverse childhood and adverse adulthood experiences with the risk of dementia and stroke among middle-aged and older adults in China.

## Introduction

As the world’s population grows and ages, the burden of noncommunicable neurological diseases continues to increase.^1^ The global prevalence of dementia is projected to rise from 57.4 million cases in 2019 to 152.8 million cases by 2050, making it the leading cause of disability and death among older adults.^2,3^ Stroke is the second leading cause of death after ischemic heart disease, accounting for 11.6% of all global deaths.^4^ The similarities in risk factors for stroke and dementia suggest shared prevention strategies.^5^ Therefore, identifying modifiable risk factors for both conditions is essential for developing integrated interventions and reducing the health care burden.

Adverse childhood experiences (ACEs) refer to traumatic exposures occurring during childhood, typically grouped into 3 categories: household dysfunction, social dysfunction, and family death or disability.^6^ ACEs may trigger toxic stress responses, leading to chronic dysregulation of the neuroendocrine-immune axis.^7,8^ ACEs have now been extensively demonstrated to be associated with multiple chronic diseases, including cardiovascular disease,^9^ depression,^10^ activities of daily living,^11^ and cognitive impairment.^12^ Traumatic exposures occurring during adulthood are defined as adverse adult experiences (AAEs), which include events such as the death of a child, lifetime discrimination, ever being confined to bed, ever being hospitalized for a month or longer, and ever leaving a job due to health conditions.^13^ These experiences are also associated with significant psychiatric sequelae, including posttraumatic stress disorder and generalized anxiety disorder.^14^ Given that AAEs may serve as a trigger for the association of ACEs with adult health outcomes, recent studies have examined their combined adverse effects on conditions such as cardiovascular disease and depression.^13,15^ However, to our knowledge, there is currently limited research exploring the independent and combined associations of ACEs and AAEs with the risk of dementia and stroke. Additionally, while there is evidence suggesting that ACEs may lead to depression,^15^ few studies have investigated whether depression plays a potential mediating role in the association of ACEs and AAEs with the risk of dementia and stroke.

Herein, we collected data on ACEs and AAEs among Chinese adults from the China Health and Retirement Longitudinal Study (CHARLS). We first examined their independent and combined associations with the incidence of dementia and stroke and further estimated the extent to which AAEs mediate the association of ACEs with incident dementia and stroke. In addition, we explored the potential interaction or joint effects of ACEs and AAEs and finally assessed whether depression mediates these associations.

## Methods

### Study Population

This cohort study used data from CHARLS, a nationally representative cohort of Chinese adults aged 45 years or older, with follow-up waves conducted from 2011 to 2020. Detailed information about the CHARLS survey has been described in previous studies.^16^ Ethical approval for collecting data on human individuals was received at the Peking University by their institutional review board, and all adults provided written informed consent. No further ethical approval was needed for using publicly available deidentifed data. This study was conducted following the Strengthening the Reporting of Observational Studies in Epidemiology (STROBE) reporting guideline.

In this study, we used data from the 2014 life course survey and the 2015 main survey of CHARLS, with baseline data collected in 2015, followed by subsequent follow-ups through 2018 and 2020 (eFigure 1 in Supplement 1 illustrates the overall study flow). Participants with ineligible age, loss to follow-up, missing ACEs and/or AAEs information, or baseline dementia or stroke were excluded (eMethods and eFigure 2 in Supplement 1). Baseline characteristics of included vs excluded participants are presented in eTable 1 in Supplement 1.

### Assessment of ACEs and AAEs

Based on prior research, we extracted 12 ACE and 5 AAE indicators from the CHARLS dataset.^6,13^ Each adverse experience was coded as a binary variable (0 = absent; 1 = present). ACEs and AAEs scores were calculated by summing the scores across all items (eTable 2 in Supplement 1). Based on participants’ ACEs and AAEs scores, we categorized them into 5 groups: 0, 1, 2, 3, or 4 and above, consistent with previous studies^6,13^ (details in the eMethods in Supplement 1). Additionally, latent class analysis (LCA) was used to further identify exposure patterns of adverse experiences. Four ACE categories (low, lower-middle risk, upper-middle risk, and high risk) and 3 AAE categories (low, medium, and high risk) were established (eFigures 3-4 in Supplement 1); the detailed procedural flow can be found in the eMethods in Supplement 1.

### Definitions of Dementia and Stroke

The outcomes of this study were new-onset dementia and stroke. Dementia was defined as the presence of both functional and cognitive impairments, or self-reported, doctor-diagnosed dementia or memory-related disorders.^17,18,19^ Meanwhile, stroke events were identified through self-reported doctor-diagnosed stroke. Detailed diagnostic procedures are provided in the eMethods in Supplement 1.

### Assessment of Depression and Covariates

Depressive symptoms were assessed in 2015 using the 10-item Centre for Epidemiologic Studies Depression Scale, with scores of 10 or greater indicating depression (eMethods and eTable 3 in Supplement 1).^20,21,22,23^ Covariates were selected based on baseline data and included age, sex, education level, marital status, smoking status, drinking status, sleep duration, diabetes, use of diabetes medication, heart disease, and cancer history (eMethods in Supplement 1).

### Statistical Analysis

Baseline characteristics are summarized as means (SDs) for continuous variables and frequencies (percentages) for categorical variables, along with corresponding 95% CIs. Missing covariates were handled as multiple imputation using the mice package in R version 4.4.2 (R Project for Statistical Computing). The proportion and number of missing values for each covariate are presented in eTable 4 in Supplement 1.

Cox proportional hazards regression analysis was used to explore the association of ACEs and AAEs with the risk of new-onset dementia and stroke, with results presented as hazard ratios (HRs) and 95% CIs. Proportional hazards assumptions were evaluated using Schoenfeld residuals, and no violations were detected (eTable 5 in Supplement 1). To assess potential multicollinearity in the fully adjusted models, generalized variance inflation factors were calculated, and no evidence of multicollinearity was observed (eTable 6 in Supplement 1). In addition, interaction analyses among covariates were conducted (details in eTables 7-8 in Supplement 1). We estimated the population attributable fractions to quantify the proportion of dementia or stroke events that could potentially be prevented if all individuals were not exposed to ACEs or AAEs. We then assessed the extent to which AAEs mediated the associations of ACEs with incident dementia and stroke. Dose-response associations were evaluated using restricted cubic spline models. Moreover, we classified participants into high- and low-risk groups using a cutoff score of 4 for both ACEs and AAEs and then created 4 joint categories to assess their combined associations with dementia and stroke. Interaction between ACEs and AAEs was further tested by adding a multiplicative term to the model. We then examined the mediating role of depression in the associations of ACEs and AAEs with incident dementia and stroke, and assessed whether health behaviors (smoking, drinking, and sleep duration) and socioeconomic status acted as significant mediators. All mediation analyses were performed using the CMAverse package in R, applying a counterfactual-based approach with 1000 bootstrap resamples for inference. To assess potential effect size modification, we conducted stratified analyses across key covariates. Several sensitivity analyses were also performed to test the robustness of the findings (eMethods in Supplement 1).

All statistical analyses were performed with R software version 4.4.2. A 2-sided *P* < .05 was set as statistical significance. Statistical analysis was performed from August 20, 2025, to November 23, 2025.

## Results

### Characteristics of the Study Population

Among 11 601 participants (mean [SD] age, 59.18 [9.41] years; 5569 male [48.0%]), 9145 (78.8%) were exposed to at least 1 ACEs indicator, 4241 (36.6%) to at least 1 AAEs indicator, and 3531 (30.4%) to both ACEs and AAEs markers (eFigure 5 in Supplement 1). Compared with individuals without stroke or dementia, those with stroke or dementia tended to be older and were more likely to be single, get less sleep, and have a higher prevalence of diabetes and heart disease, along with higher AAEs and ACEs scores (Table 1 and eTable 9 in Supplement 1).

**Table 1.** Baseline Characteristics of the Study Population

Characteristic	Participants, No. (%)						
	Overall (N = 11 601)	No dementia (n = 11 143)	Dementia (n = 458)	*P* value^a^	No stroke (n = 10 824)	Stroke (n = 777)	*P* value^a^
Age, mean (SD), y	59.18 (9.41)	58.92 (9.31)	65.51 (9.56)	<.001	58.94 (9.41)	62.45 (8.75)	<.001
Sex							
Female	6032 (52.0)	5763 (51.7)	269 (58.7)	.003	5625 (52.0)	407 (52.4)	.82
Male	5569 (48.0)	5380 (48.3)	189 (41.3)		5199 (48.0)	370 (47.6)	
Marital status							
Married	10 255 (88.4)	9888 (88.7)	367 (80.1)	<.001	9593 (88.6)	662 (85.2)	.004
Single	1346 (11.6)	1255 (11.3)	91 (19.9)		1231 (11.4)	115 (14.8)	
Education							
Above high school	369 (3.2)	355 (3.2)	14 (3.1)	<.001	343 (3.2)	26 (3.3)	.14
Secondary school	3247 (28.0)	3163 (28.4)	84 (18.3)		3049 (28.2)	198 (25.5)	
Primary	5168 (44.5)	4959 (44.5)	209 (45.6)		4828 (44.6)	340 (43.8)	
No formal education	2817 (24.3)	2666 (23.9)	151 (33.0)		2604 (24.1)	213 (27.4)	
Drinking							
No	6162 (53.1)	5912 (53.1)	250 (54.6)	.52	5739 (53.0)	423 (54.4)	.44
Yes	5439 (46.9)	5231 (46.9)	208 (45.4)		5085 (47.0)	354 (45.6)	
Smoking							
No	6556 (56.5)	6277 (56.3)	279 (60.9)	.05	6133 (56.7)	423 (54.4)	.23
Yes	5045 (43.5)	4866 (43.7)	179 (39.1)		4691 (43.3)	354 (45.6)	
Sleep, h/d							
≤6	5732 (49.4)	5478 (49.2)	254 (55.5)	.008	5313 (49.1)	419 (53.9)	.009
>6	5869 (50.6)	5665 (50.8)	204 (44.5)		5511 (50.9)	358 (46.1)	
Diabetes							
No	10 521 (90.7)	10 137 (91.0)	384 (83.8)	<.001	9876 (91.2)	645 (83.0)	<.001
Yes	1080 (9.3)	1006 (9.0)	74 (16.2)		948 (8.8)	132 (17.0)	
Heart disease							
No	9674 (83.4)	9359 (84.0)	315 (68.8)	<.001	9127 (84.3)	547 (70.4)	<.001
Yes	1927 (16.6)	1784 (16.0)	143 (31.2)		1697 (15.7)	230 (29.6)	
Cancer history							
No	11 414 (98.4)	10 969 (98.4)	445 (97.2)	.03	10 647 (98.4)	767 (98.7)	.46
Yes	187 (1.6)	174 (1.6)	13 (2.8)		177 (1.6)	10 (1.3)	
ACEs score, mean (SD)	1.70 (1.41)	1.69 (1.41)	1.98 (1.55)	<.001	1.70 (1.41)	1.81 (1.46)	.05
ACEs category							
0	2456 (21.2)	2382 (21.4)	74 (16.2)	.002	2305 (21.3)	151 (19.4)	.08
1	3459 (29.8)	3327 (29.9)	132 (28.8)		3228 (29.8)	231 (29.7)	
2	2757 (23.8)	2651 (23.8)	106 (23.1)		2587 (23.9)	170 (21.9)	
3	1644 (14.2)	1570 (14.1)	74 (16.2)		1526 (14.1)	118 (15.2)	
≥4	1285 (11.1)	1213 (10.9)	72 (15.7)		1178 (10.9)	107 (13.8)	
AAEs score, mean (SD)	0.64 (1.01)	0.62 (1.00)	1.00 (1.19)	<.001	0.62 (1.00)	0.90 (1.20)	<.001
AAEs category							
0	7360 (63.4)	7152 (64.2)	208 (45.4)	<.001	6949 (64.2)	411 (52.9)	<.001
1	2258 (19.5)	2132 (19.1)	126 (27.5)		2089 (19.3)	169 (21.8)	
2	1083 (9.3)	1020 (9.2)	63 (13.8)		985 (9.1)	98 (12.6)	
3	652 (5.6)	611 (5.5)	41 (9.0)		587 (5.4)	65 (8.4)	
≥4	248 (2.1)	228 (2.0)	20 (4.4)		214 (2.0)	34 (4.4)	

Abbreviations: AAEs, adverse adulthood experiences; ACEs, adverse childhood experiences.

^a^ Continuous variables were compared using the *t* test or Wilcoxon rank sum test.

### Associations of ACEs and AAEs With Incident Dementia and Stroke

During a mean (SD) follow-up of 4.89 (0.48) years for dementia and 4.84 (0.57) years for stroke, 458 dementia events and 777 stroke events occurred. After fully adjusting for covariates, higher ACEs (HR, 1.11; 95% CI, 1.05-1.18) and AAEs (HR, 1.23; 95% CI, 1.14-1.33) were associated with higher hazards of dementia during follow-up, assuming proportional hazards (Table 2). Furthermore, when ACEs and AAEs were examined as 5 categories, compared with individuals with no adverse experiences, those with exposure to 4 or more ACEs and 4 or more AAEs had higher hazards of dementia (ACEs: HR, 1.64; 95% CI, 1.18-2.27; AAEs: HR, 2.41; 95% CI, 1.52-3.83). For stroke, higher AAEs scores were positively associated with stroke hazards (HR, 1.19; 95% CI, 1.12-1.26), and individuals with scores of 4 or greater had a 125% higher hazard of stroke during follow-up (HR, 2.25; 95% CI, 1.58-3.20) (Table 3). However, no association of ACEs with stroke incidence was found in the final model.

**Table 2.** Associations of ACEs and AAEs With the Risk of Dementia

Exposure	Participants, No.	No. of cases per person-years	Model 1: Dementia, HR (95% CI)^a^	*P* value	Model 2: Dementia, HR (95% CI)^b^	*P* value	Model 3: Dementia, HR (95% CI)^c^	*P* value	PAF, % (95% CI)
ACEs score	11 601	NA	1.14 (1.07-1.21)	<.001	1.13 (1.06-1.20)	<.001	1.11 (1.05-1.18)	.001	NA
ACEs category	NA	NA	NA	NA	NA	NA	NA		23.68 (8.01-39.35)
0	2456	74/12 030	1 [Reference]	NA	1 [Reference]	NA	1 [Reference]	NA	NA
1	3459	132/16 912	1.27 (0.95-1.69)	.10	1.20 (0.90-1.60)	.20	1.18 (0.89-1.57)	.25	NA
2	2757	106/13 475	1.28 (0.95-1.72)	.10	1.21 (0.90-1.63)	.21	1.15 (0.85-1.55)	.36	NA
3	1644	74/8043	1.50 (1.09-2.07)	.01	1.46 (1.06-2.02)	.02	1.36 (0.99-1.89)	.06	NA
≥4	1285	72/6272	1.85 (1.34-2.56)	<.001	1.74 (1.26-2.41)	.001	1.64 (1.18-2.27)	.003	NA
*P* value for trend^d^	NA	NA	NA	<.001	NA	<.001	NA	.003	NA
AAEs score	11 601	NA	1.34 (1.25-1.44)	<.001	1.27 (1.18-1.37)	<.001	1.23 (1.14-1.33)	<.001	NA
AAEs category		NA	NA	NA	NA	NA	NA		28.42 (21.36-35.47)
0	7360	208/36 253	1 [Reference]	NA	1 [Reference]	NA	1 [Reference]	NA	NA
1	2258	126/10 928	2.04 (1.63-2.54)	<.001	1.58 (1.26-1.98)	<.001	1.54 (1.22-1.93)	<.001	NA
2	1083	63/5231	2.13 (1.61-2.82)	<.001	1.74 (1.31-2.31)	<.001	1.60 (1.20-2.13)	.001	NA
3	652	41/3141	2.28 (1.63-3.19)	<.001	1.93 (1.38-2.71)	<.001	1.76 (1.26-2.48)	.001	NA
≥4	248	20/1181	3.01 (1.90-4.77)	<.001	2.67 (1.68-4.22)	<.001	2.41 (1.52-3.83)	<.001	NA
*P* value for trend^d^	NA	NA	NA	<.001	NA	<.001	NA	<.001	NA

Abbreviations: AAEs, adverse adulthood experiences; ACEs, adverse childhood experiences; HR, hazard ratio; NA, not applicable; PAF, population attributable fractions.

^a^ Model 1 was unadjusted for any covariates.

^b^ Model 2 was adjusted for age and sex.

^c^ Model 3 was further adjusted for education, marital status, smoking status, drinking status, sleep, diabetes, diabetes medication, heart disease, and cancer history based on Model 2.

^d^ *P* value for trend was calculated by modeling the categorical variable as an ordinal continuous variable in the Cox regression.

**Table 3.** Associations of ACEs and AAEs With the Risk of Stroke

Exposure	Participants, No.	No. of cases per person-years	Model 1: Stroke, HR (95% CI)^a^	*P* value	Model 2: Stroke, HR (95% CI)^b^	*P* value	Model 3: Stroke, HR (95% CI)^c^	*P* value	PAF, % (95% CI)
ACEs score	11 601	NA	1.05 (1.00 to 1.10)	.04	1.05 (1.00 to 1.10)^d^	.07	1.04 (0.99 to 1.09)	.14	NA
ACEs category	NA	NA	NA	NA	NA	NA	NA	NA	8.20 (−4.52 to 20.93)
0	2456	151/11 898	1 [Reference]	NA	1 [Reference]	NA	1 [Reference]	NA	NA
1	3459	231/16 719	1.09 (0.89 to 1.34)	.41	1.05 (0.86 to 1.29)	.62	1.05 (0.85 to 1.29)	.66	NA
2	2757	170/13 353	1.00 (0.81 to 1.25)	.97	0.96 (0.77 to 1.20)	.74	0.93 (0.75 to 1.16)	.54	NA
3	1644	118/7940	1.18 (0.92 to 1.50)	.19	1.14 (0.90 to 1.46)	.27	1.10 (0.87 to 1.41)	.43	NA
≥4	1285	107/6221	1.35 (1.06 to 1.73)	.02	1.29 (1.01 to 1.66)	.04	1.26 (0.98 to 1.62)	.07	NA
*P* value for trend^e^	NA	NA	NA	.03	NA	.06	NA	.12	NA
AAEs score	11 601		1.26 (1.19 to 1.34)	<.001	1.22 (1.15 to 1.29)	<.001	1.19 (1.12 to 1.26)	<.001	NA
AAEs category	NA	NA	NA	NA	NA	NA	NA	NA	16.62 (11.28 to 21.96)
0	7360	411/35 883	1 [Reference]	NA	1 [Reference]	NA	1 [Reference]	NA	NA
1	2258	169/10 826	1.38 (1.15 to 1.65)	<.001	1.21 (1.01 to 1.46)	.04	1.20 (1.00 to 1.44)	.05	NA
2	1083	98/5169	1.67 (1.34 to 2.09)	<.001	1.50 (1.21 to 1.88)	<.001	1.40 (1.12 to 1.74)	.003	NA
3	652	65/3091	1.85 (1.42 to 2.40)	<.001	1.67 (1.28 to 2.17)	<.001	1.52 (1.17 to 1.98)	.002	NA
≥4	248	34/1163	2.61 (1.84 to 3.70)	<.001	2.44 (1.72 to 3.46)	<.001	2.25 (1.58 to 3.20)	<.001	NA
*P* value for trend^e^	NA	NA	NA	<.001	NA	<.001	NA	<.001	NA

Abbreviations: AAEs, adverse adulthood experiences; ACEs, adverse childhood experiences; HR, hazard ratio; NA, not applicable; PAF, population attributable fractions.

^a^ Model 1 was unadjusted for any covariates.

^b^ Model 2 was adjusted for age and sex.

^c^ Model 3 was further adjusted for education, marital status, smoking status, drinking status, sleep, diabetes, diabetes medication, heart disease, and cancer history based on Model 2.

^d^ CI rounded to 1.00 (original value, 0.997).

^e^ *P* value for trend was calculated by modeling the categorical variable as an ordinal continuous variable in the Cox regression.

**Figure 1.**
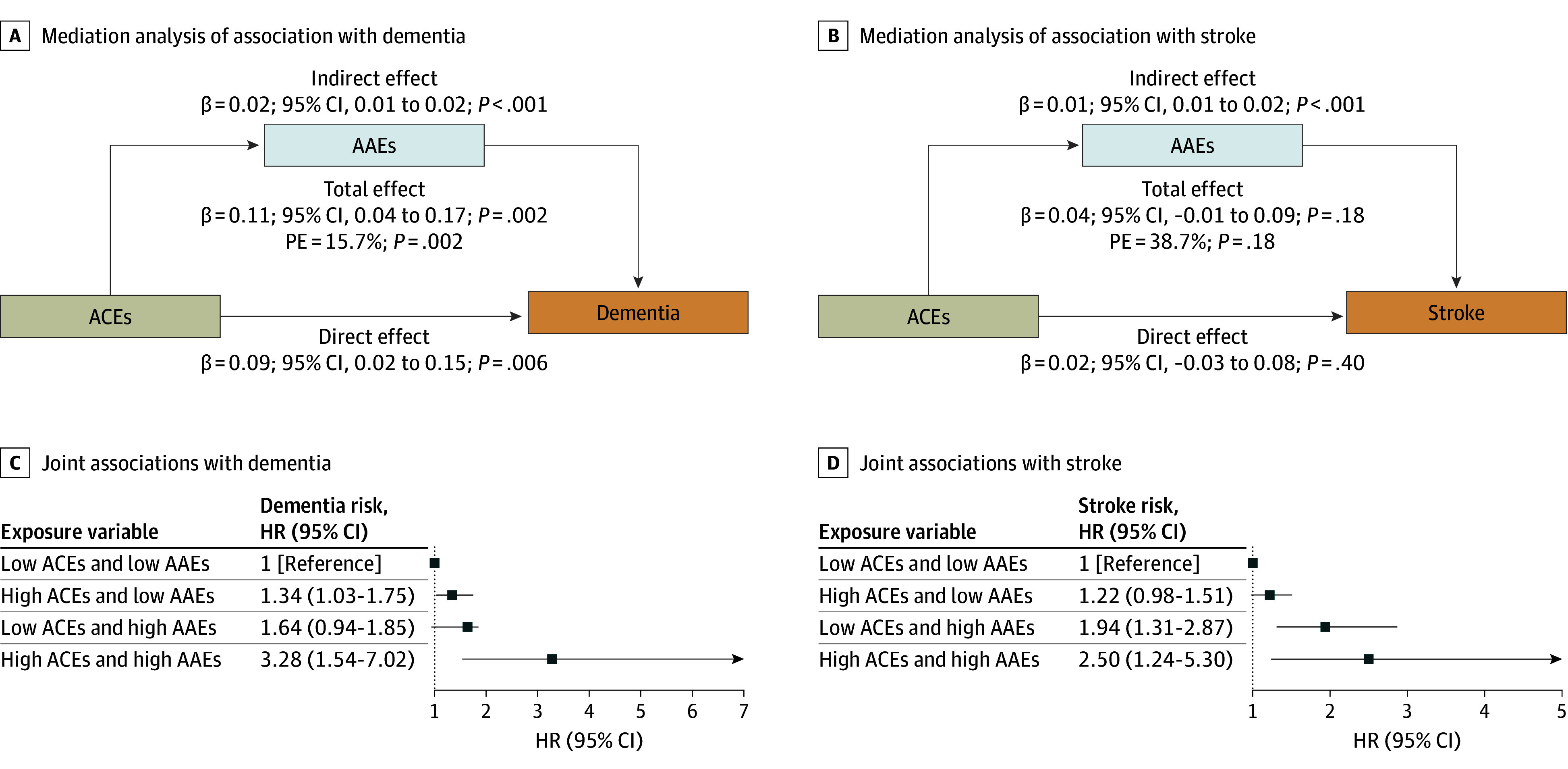
Mediation and Joint Analyses of Adverse Childhood Experiences (ACEs) and Adverse Adulthood Experiences (AAEs) With the Risk of Dementia and Stroke The analysis was performed based on Model 3 (adjusted for age, sex, education, marital status, smoking status, drinking status, sleep, diabetes, diabetes medication, heart disease, and cancer history). HR indicates hazard ratio; PE, proportion of mediation.

In the association of overall ACEs with incident dementia, AAEs accounted for 15.7% of the mediating effect (β = 0.11; 95% CI, 0.04 to 0.17; *P* = .002), whereas in the association with incident stroke, AAEs accounted for 38.7% of the mediating effect (β = 0.04; 95% CI, −0.01 to 0.09; *P* = .18), although this was not statistically significant (Figure 1 A,B). Moreover, the restricted cubic spline curves showed no evidence of nonlinear associations (eFigure 6 in Supplement 1), and significant positive dose-response associations were observed for ACEs with dementia (*P* = .002) as well as for AAEs with dementia (*P* < .001) and stroke (*P* < .001).

In further analysis of the association of ACE and AAE classes identified through LCA with the outcomes of interest, we found that the high ACEs and high AAEs classes were associated with 60% (HR, 1.60; 95% CI, 1.23-2.08) and 51% (HR, 1.51; 95% CI, 1.07-2.15) higher hazards of dementia, respectively, and 33% (HR, 1.33; 95% CI, 1.08-1.65) and 45% (HR, 1.45; 95% CI, 1.10-1.92) higher hazards of stroke, respectively (eTables 10-11 in Supplement 1).

When ACEs and AAEs were dichotomized at a cutoff of 4, compared with the low-risk group, the high-risk ACEs group showed 40% (HR, 1.40; 95% CI, 1.09-1.81) and 24% (HR, 1.24; 95% CI, 1.01-1.52) higher hazards of dementia and stroke, respectively (eTables 12-13 in Supplement 1). For the high-risk AAEs group, the corresponding increases were 91% (HR, 1.91; 95% CI, 1.21-2.99) for dementia and 99% (HR, 1.99; 95% CI, 1.41-2.82) for stroke. When examining the joint effects of dichotomized ACEs and AAEs, participants in the high-risk groups for both ACEs and AAEs exhibited 228% (HR, 3.28; 95% CI, 1.54-7.02) and 150% (HR, 2.50; 95% CI, 1.24-5.30) higher hazards of dementia and stroke, respectively, compared with the low-risk group (Figure 1 C,D; eTable 14 in Supplement 1). These hazards were higher than those for individuals exposed to high ACEs or high AAEs alone. No significant interaction between ACEs and AAEs was observed.

### Mediation Analysis

**Figure 2.**
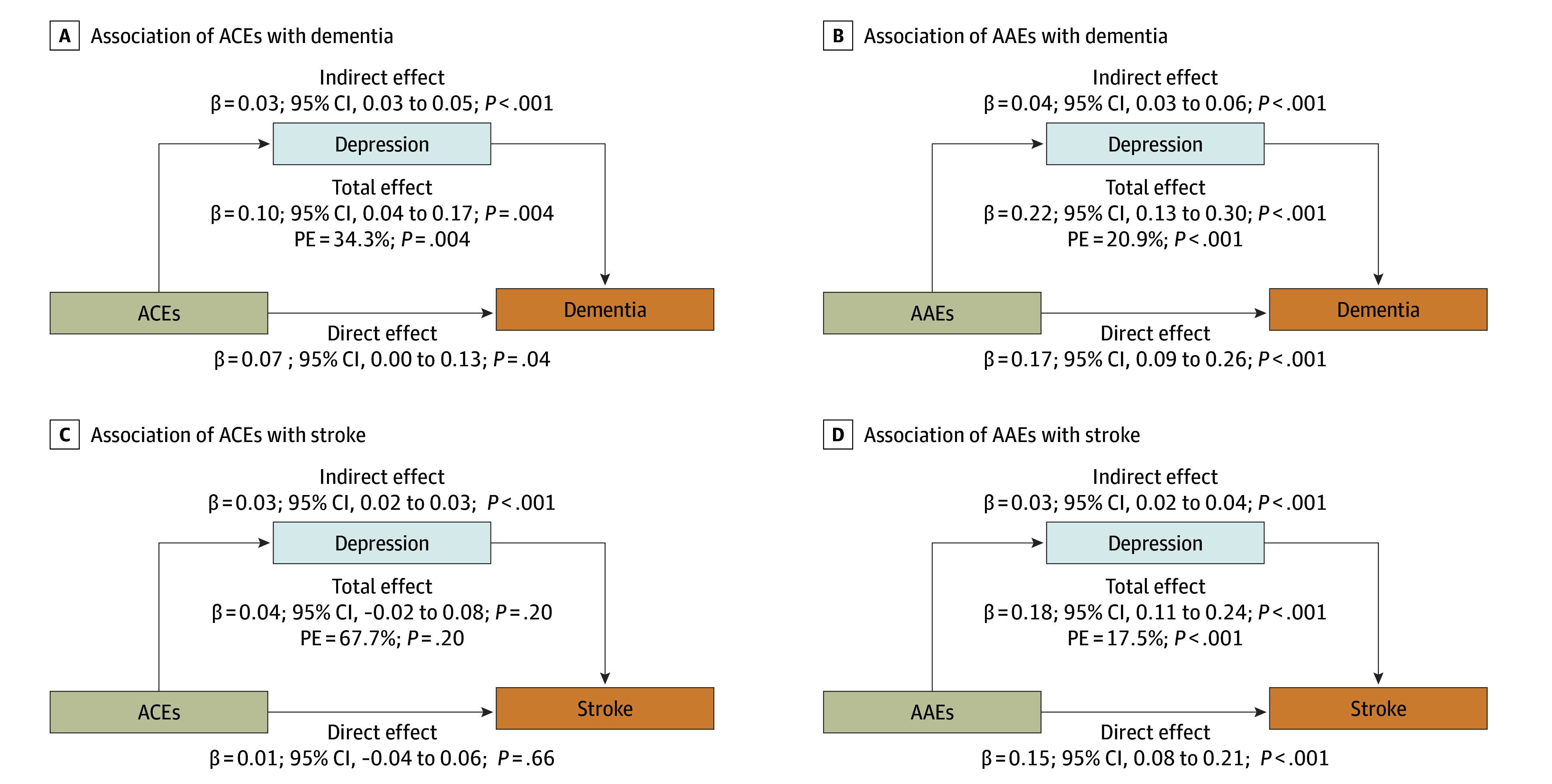
Mediation Analysis of Depression on Associations of Adverse Childhood Experiences (ACEs) and Adverse Adulthood Experiences (AAEs) With Incident Dementia and Stroke The analysis was performed in Model 3 (adjusted for age, sex, education, marital status, smoking status, drinking status, sleep, diabetes, diabetes medication, heart disease, and cancer history). PE indicates proportion of mediation.

Figure 2 illustrates the mediating role of depression in the associations of ACEs and AAEs with incident dementia and stroke. In the multivariable-adjusted model, depression was found to mediate the associations of ACEs with dementia (mediation proportion, 34.3%; β = 0.10; 95% CI, 0.04-0.17; *P* = .004), AAEs with dementia (mediation proportion, 20.9%; β = 0.22; 95% CI, 0.13-0.30; *P* < .001), and AAEs with stroke (mediation proportion, 17.5%; β = 0.18; 95% CI, 0.11-0.24; *P* < .001). No mediating effect was observed for the association of ACEs with stroke. Additional mediation analyses revealed no significant mediating effects for other variables, including health behaviors and socioeconomic status (eFigures 7-10 in Supplement 1).

### Subgroup and Sensitivity Analyses

Subgroup and sensitivity analyses found that sex modified the association of AAEs with dementia, with higher hazards observed in male participants (HR, 1.35; 95% CI, 1.21-1.51) than in females (HR, 1.15; 95% CI, 1.03-1.28) (*P* for interaction = .049). Likewise, the association of AAEs with stroke was more pronounced in participants younger than 65 years (HR, 1.24; 95% CI, 1.15-1.34) compared with those aged 65 years or older (HR, 1.11; 95% CI, 1.01-1.23) (*P* for interaction = .02) (eFigures 11-12 and eTables 15-18 in Supplement 1).

Further analysis of individual ACE and AAE components revealed that 3 ACE indicators (childhood household mental illness, domestic violence, and parental disability) were associated with dementia, while 3 AAE indicators (ever confined to bed, hospitalized ≥1 month, and leaving a job due to health problems) were associated with both dementia and stroke (eTables 19-20 in Supplement 1). Additionally, all associations remained robust across multiple sensitivity analyses, including complete-case analysis, exclusion of baseline cancer history, stricter dementia definition, competing-risk models, further adjustment for socioeconomic status, parental education and residential type, adjustment for biomarkers in complete cases, and inclusion of covariate interaction terms (eTables 21-33 in Supplement 1).

## Discussion

This cohort study found that higher exposure to either ACEs or AAEs was associated with dementia and stroke incidence, with the highest risk observed in the combined high-risk group. LCA was employed to better identify distinct patterns of exposure to ACEs or AAEs and their corresponding risk groups, thereby complementing the limitations of the original classification. Furthermore, depression partially mediated these associations. Subgroup analyses indicated that the association of AAEs with dementia was more pronounced in men, whereas the association of AAEs with stroke was more pronounced in younger individuals.

Our study found that the association of ACEs and AAEs with dementia is consistent with previous research.^24^ Recent studies suggest that ACEs may increase the incidence of dementia by influencing brain development and triggering compensatory neural responses associated with aging.^8,25,26^ Additionally, AAEs, as a newly proposed indicator, have recently been shown to be associated with an increased risk of depression and cardiovascular disease.^13,15^ Our study further extends previous findings by demonstrating that AAEs are associated with incident dementia and stroke. Of note, LCA identified a high-risk subgroup that demonstrated a statistically significant association with stroke risk, which was not detected using the conventional count-based approach. This finding suggests that LCA is able to uncover latent subpopulations with distinct patterns of adverse childhood exposures that may remain obscured under traditional scoring methods.^27,28^ This enhanced sensitivity helps reveal subtle associations—such as that between ACEs and stroke risk in specific subgroups—that might otherwise be overlooked. Furthermore, identifying these distinct risk profiles supports population health management by enabling more targeted interventions for high-risk subgroups and optimizing the allocation of preventive resources.^29^

We found that simultaneous exposure to both high-risk ACEs and AAEs factors significantly increased the risk of stroke and dementia, suggesting that ACEs and AAEs may interact to amplify their effects. Specifically, relatively early life adverse experiences may foster erroneous cognitive biases and a heightened sense of threat related to stress. This, in turn, increases the likelihood of encountering more substantial stressors in adulthood and reduces adaptability to these stressors, thereby exerting greater harm to health.^30,31^ Moreover, our study indicated that depression mediated the associations of ACEs and AAEs with incident dementia and the association of AAEs with incident stroke. Previous research has shown that depression is more prevalent among individuals exposed to both ACEs and AAEs,^15^ and that the impact of ACEs on cognitive function is largely transmitted through depressive symptoms.^32^ Our findings further underscore the pivotal role of depression as a pathway linking early life and adult-life adversities to later-life neurological outcomes, highlighting the need for interventions aimed at preventing ACEs and AAEs across the life course and managing depressive symptoms to reduce the risks of dementia and stroke.

Subgroup analyses revealed that the association of AAEs with dementia and stroke was more pronounced in men and younger individuals. Research indicates that certain adverse events constitute greater risk factors for dementia in men, whereas this association is reduced in women.^33^ Our research suggests that such gender disparities may stem from unique biopsychosocial factors specific to men and women. For instance, within China’s historical context, men may bear greater economic burdens, are more likely to engage in long-hour full-time employment, and consequently have reduced time for social activities that promote relaxation. This reduction in social engagement may in turn increase the risk of cognitive decline and dementia.^33^ In younger adults, exposure to AAEs may exert larger effects on stroke risk because their physiological and psychological systems have not yet fully adapted to chronic stressors. Compared with older adults, younger individuals exhibit greater hypothalamic-pituitary-adrenal axis reactivity and heightened inflammatory responses, reflecting limited stress adaptation.^34,35^ This maladaptive response makes AAEs more predictive of stroke risk in younger populations. Moreover, younger adults may be more vulnerable to psychological consequences such as depression, which can accelerate the onset of stroke events.^36^ However, further research is needed to confirm their exact mechanisms.

### Strengths and Limitations

The strengths of this study lie in its large sample size and strong representativeness. However, several limitations should also be acknowledged. First, dementia is not diagnosed by neurologists; however, this algorithmic definition is based on standard practice within population-based cohorts and has been validated through clinical diagnostic surveys and multiple studies.^17,18,37^ Second, information on ACEs and AAEs relies on retrospective self-report, which may be susceptible to reporting and recall biases. Nevertheless, prior research has demonstrated good test-retest reliability for retrospective measures of ACEs.^38^ Third, this study utilized physician-reported diagnoses, which may underestimate the incidence of stroke events. Fourth, baseline differences between included and excluded participants may, to some extent, introduce selection bias. Fifth, depression may act as a prodromal symptom of dementia; hence, despite using baseline depression status, residual reverse causation may still be present in the association of depression with dementia. Sixth, despite controlling for multiple covariates and accounting for various biomarkers in sensitivity analyses, residual confounding may still exist, and causality cannot be confirmed.

## Conclusions

In this cohort study of middle-aged and older adults, exposure to ACEs or AAEs was associated with increased risks of dementia and stroke, and depression partly mediated these associations. These findings underscore the importance of addressing life-course psychosocial stress and implementing targeted depression prevention among high-risk groups to reduce the burden of dementia and stroke.

## Notes

Supplement 1. eFigure 1. Overview of study design and key analyses eMethods. eFigure 2. Flowchart of the included population eTable 1. Baseline characteristics of included and excluded participants eTable 2. Questionnaire items and responses of each ACEs and AAEs indicator eFigure 3. Distribution of item-response probabilities across latent classes of ACEs eFigure 4. Distribution of item-response probabilities across latent classes of AAEs eTable 3. The items of the Center for Epidemiological Survey Depression Scale eTable 4. Missing data proportions for covariates eTable 5. Summary of Cox proportional hazards assumption tests eTable 6. GVIF-based multicollinearity diagnostics for covariates included in Model 3 eTable 7. Interaction analyses among covariates included in Model 3 for associations of ACEs and AAEs with incident dementia eTable 8. Interaction analyses among covariates included in Model 3 for associations of ACEs and AAEs with incident stroke eFigure 5. Venn diagram of the study population by exposure status eTable 9. Baseline characteristics of study participants with 95% CI for OR (categorical variables) and mean differences (continuous variables) eFigure 6. RCS analyses of the associations of ACEs (A-B) and AAEs (C-D) with the risk of dementia and stroke eTable 10. Associations of latent ACEs and AAEs classes with incident dementia eTable 11. Associations of latent ACEs and AAEs classes with incident stroke eTable 12. Associations of ACEs-binary and AAEs-binary groups with incident dementia eTable 13. Associations of ACEs-binary and AAEs-binary groups with incident stroke eTable 14. Joint analyses of ACEs and AAEs with incident dementia and stroke eFigure 7. Mediation analysis of smoking on associations of ACEs and AAEs with incident dementia and stroke eFigure 8. Mediation analysis of drinking on associations of ACEs and AAEs with incident dementia and stroke eFigure 9. Mediation analysis of sleep on associations of ACEs and AAEs with incident dementia and stroke eFigure 10. Mediation analysis of SES on associations of ACEs and AAEs with incident dementia and stroke eFigure 11. Subgroup analysis of the associations of ACEs and AAEs with incident dementia eFigure 12. Subgroup analysis of the associations of ACEs and AAEs with incident stroke eTable 15. Subgroup analysis of the associations of ACEs and AAEs with incident dementia eTable 16. Subgroup analysis of the associations of ACEs and AAEs with incident stroke eTable 17. Sensitivity and subgroup analyses of the associations of ACEs and AAEs with incident dementia, stratified by SES, parental education, and residential type eTable 18. Sensitivity and subgroup analyses of the associations of ACEs and AAEs with incident stroke, stratified by SES, parental education, and residential type eTable 19. Associations of individual components of ACEs and AAEs with incident dementia eTable 20. Associations of individual components of ACEs and AAEs with incident stroke eTable 21. Sensitivity analyses for associations of ACEs and AAEs with incident dementia excluded all samples with missing covariates (N = 9360) eTable 22. Sensitivity analyses for associations of ACEs and AAEs with incident stroke excluded all samples with missing covariates (N = 9360) eTable 23. Sensitivity analyses for associations of ACEs and AAEs with incident dementia excluded participants with diagnosed as cancer history at baseline (N = 11 414) eTable 24. Sensitivity analyses for associations of ACEs and AAEs with incident stroke excluded participants with diagnosed as cancer history at baseline (N = 11 414) eTable 25. Sensitivity analyses for associations of ACEs and AAEs with incident dementia, redefining dementia strictly by cognitive impairment and ADL limitations (N = 11 282) eTable 26. Sensitivity analyses for associations of ACEs and AAEs with incident dementia using competing risk model eTable 27. Sensitivity analyses for associations of ACEs and AAEs with incident stroke using competing risk model eTable 28. Sensitivity analyses for associations of ACEs and AAEs with incident dementia additionally adjusted for SES, parental education and residential type eTable 29. Sensitivity analyses for associations of ACEs and AAEs with incident stroke additionally adjusted for SES, parental education and residential type eTable 30. Sensitivity analyses for associations of ACEs and AAEs with incident dementia additionally adjusted for biomarker (N = 8006) eTable 31. Sensitivity analyses for associations of ACEs and AAEs with incident stroke additionally adjusted for biomarker (N = 8006) eTable 32. Sensitivity analyses for associations of ACEs and AAEs with incident dementia additionally including interaction terms between covariates eTable 33. Sensitivity analyses for associations of ACEs and AAEs with incident stroke additionally including interaction terms between covariates eReferences

Supplement 2. Data Sharing Statement
