# Supplementary material for: Life-Course Psychosocial Stress and Risk of Dementia and Stroke in Middle-Aged and Older Adults
Source: JAMA Netw Open. 2026 Jan 28;9(1):e2556012. doi: 10.1001/jamanetworkopen.2025.56012 (PMC12853204; doi:10.1001/jamanetworkopen.2025.56012)
Supplement: Supplement 1. — eFigure 1. Overview of study design and key analyses eMethods. eFigure 2. Flowchart of the included population eTable 1. Baseline characteristics of included and excluded participants eTable 2. Questionnaire items and responses of each ACEs and AAEs indicator eFigure 3. Distribution of item-response probabilities across latent classes of ACEs eFigure 4. Distribution of item-response probabilities across latent classes of AAEs eTable 3. The items of the Center for Epidemiological Survey Depression Scale eTable 4. Missing data proportions for covariates eTable 5. Summary of Cox proportional hazards assumption tests eTable 6. GVIF-based multicollinearity diagnostics for covariates included in Model 3 eTable 7. Interaction analyses among covariates included in Model 3 for associations of ACEs and AAEs with incident dementia eTable 8. Interaction analyses among covariates included in Model 3 for associations of ACEs and AAEs with incident stroke eFigure 5. Venn diagram of the study population by exposure status eTable 9. Baseline characteristics of study participants with 95% CI for OR (categorical variables) and mean differences (continuous variables) eFigure 6. RCS analyses of the associations of ACEs (A-B) and AAEs (C-D) with the risk of dementia and stroke eTable 10. Associations of latent ACEs and AAEs classes with incident dementia eTable 11. Associations of latent ACEs and AAEs classes with incident stroke eTable 12. Associations of ACEs-binary and AAEs-binary groups with incident dementia eTable 13. Associations of ACEs-binary and AAEs-binary groups with incident stroke eTable 14. Joint analyses of ACEs and AAEs with incident dementia and stroke eFigure 7. Mediation analysis of smoking on associations of ACEs and AAEs with incident dementia and stroke eFigure 8. Mediation analysis of drinking on associations of ACEs and AAEs with incident dementia and stroke eFigure 9. Mediation analysis of sleep on associations of ACEs and AAEs with incident dementia and stroke [file jamanetwopen-e2556012-s001.pdf]

Supplemental Online Content

Chen B, Xue E, Li Y, et al. Life-course psychosocial stress and risk of dementia and stroke in middle-aged and older adults. *JAMA Netw. Open*. 2026;9(1):e2556012. doi:10.1001/jamanetworkopen.2025.56012

eFigure 1. Overview of study design and key analyses

eMethods.

eFigure 2. Flowchart of the included population

eTable 1. Baseline characteristics of included and excluded participants

eTable 2. Questionnaire items and responses of each ACEs and AAEs indicator

eFigure 3. Distribution of item-response probabilities across latent classes of ACEs

eFigure 4. Distribution of item-response probabilities across latent classes of AAEs

eTable 3. The items of the Center for Epidemiological Survey Depression Scale

eTable 4. Missing data proportions for covariates

eTable 5. Summary of Cox proportional hazards assumption tests

eTable 6. GVIF-based multicollinearity diagnostics for covariates included in Model 3

eTable 7. Interaction analyses among covariates included in Model 3 for associations of ACEs and AAEs with incident dementia

eTable 8. Interaction analyses among covariates included in Model 3 for associations of ACEs and AAEs with incident stroke

eFigure 5. Venn diagram of the study population by exposure status

eTable 9. Baseline characteristics of study participants with 95% CI for OR (categorical variables) and mean differences (continuous variables)

eFigure 6. RCS analyses of the associations of ACEs (A-B) and AAEs (C-D) with the risk of dementia and stroke

eTable 10. Associations of latent ACEs and AAEs classes with incident dementia

eTable 11. Associations of latent ACEs and AAEs classes with incident stroke

eTable 12. Associations of ACEs-binary and AAEs-binary groups with incident dementia

eTable 13. Associations of ACEs-binary and AAEs-binary groups with incident stroke

eTable 14. Joint analyses of ACEs and AAEs with incident dementia and stroke

eFigure 7. Mediation analysis of smoking on associations of ACEs and AAEs with incident dementia and stroke

eFigure 8. Mediation analysis of drinking on associations of ACEs and AAEs with incident dementia and stroke

eFigure 9. Mediation analysis of sleep on associations of ACEs and AAEs with incident dementia and stroke

eFigure 10. Mediation analysis of SES on associations of ACEs and AAEs with incident dementia and stroke

eFigure 11. Subgroup analysis of the associations of ACEs and AAEs with incident dementia

eFigure 12. Subgroup analysis of the associations of ACEs and AAEs with incident stroke

eTable 15. Subgroup analysis of the associations of ACEs and AAEs with incident dementia

eTable 16. Subgroup analysis of the associations of ACEs and AAEs with incident stroke

© 2026 Chen B et al. *JAMA Network Open*.

eTable 17. Sensitivity and subgroup analyses of the associations of ACEs and AAEs with incident dementia, stratified by SES, parental education, and residential type

eTable 18. Sensitivity and subgroup analyses of the associations of ACEs and AAEs with incident stroke, stratified by SES, parental education, and residential type

eTable 19. Associations of individual components of ACEs and AAEs with incident dementia

eTable 20. Associations of individual components of ACEs and AAEs with incident stroke

eTable 21. Sensitivity analyses for associations of ACEs and AAEs with incident dementia excluded all samples with missing covariates (N = 9360)

eTable 22. Sensitivity analyses for associations of ACEs and AAEs with incident stroke excluded all samples with missing covariates (N = 9360)

eTable 23. Sensitivity analyses for associations of ACEs and AAEs with incident dementia excluded participants with diagnosed as cancer history at baseline (N = 11 414)

eTable 24. Sensitivity analyses for associations of ACEs and AAEs with incident stroke excluded participants with diagnosed as cancer history at baseline (N = 11 414)

eTable 25. Sensitivity analyses for associations of ACEs and AAEs with incident dementia, redefining dementia strictly by cognitive impairment and ADL limitations (N = 11 282)

eTable 26. Sensitivity analyses for associations of ACEs and AAEs with incident dementia using competing risk model

eTable 27. Sensitivity analyses for associations of ACEs and AAEs with incident stroke using competing risk model

eTable 28. Sensitivity analyses for associations of ACEs and AAEs with incident dementia additionally adjusted for SES, parental education and residential type

© 2026 Chen B et al. *JAMA Network Open*.

eTable 29. Sensitivity analyses for associations of ACEs and AAEs with incident stroke additionally adjusted for SES, parental education and residential type

eTable 30. Sensitivity analyses for associations of ACEs and AAEs with incident dementia additionally adjusted for biomarker (N = 8006)

eTable 31. Sensitivity analyses for associations of ACEs and AAEs with incident stroke additionally adjusted for biomarker (N = 8006)

eTable 32. Sensitivity analyses for associations of ACEs and AAEs with incident dementia additionally including interaction terms between covariates

eTable 33. Sensitivity analyses for associations of ACEs and AAEs with incident stroke additionally including interaction terms between covariates

eReferences

This supplemental material has been provided by the authors to give readers additional information about their work.

eFigure 1. Overview of study design and key analyses

Higher exposures to ACEs and AAEs were each associated with increased risks of dementia and stroke, with the joint high-risk group showing the greatest risk elevation. Depression partially mediated these associations. AAEs indicates adverse adulthood experiences; ACEs, adverse childhood experiences; CI, confidence interval; HR, hazard ratio; and PE, proportion of mediation.

© 2026 Chen B et al. *JAMA Network Open*.

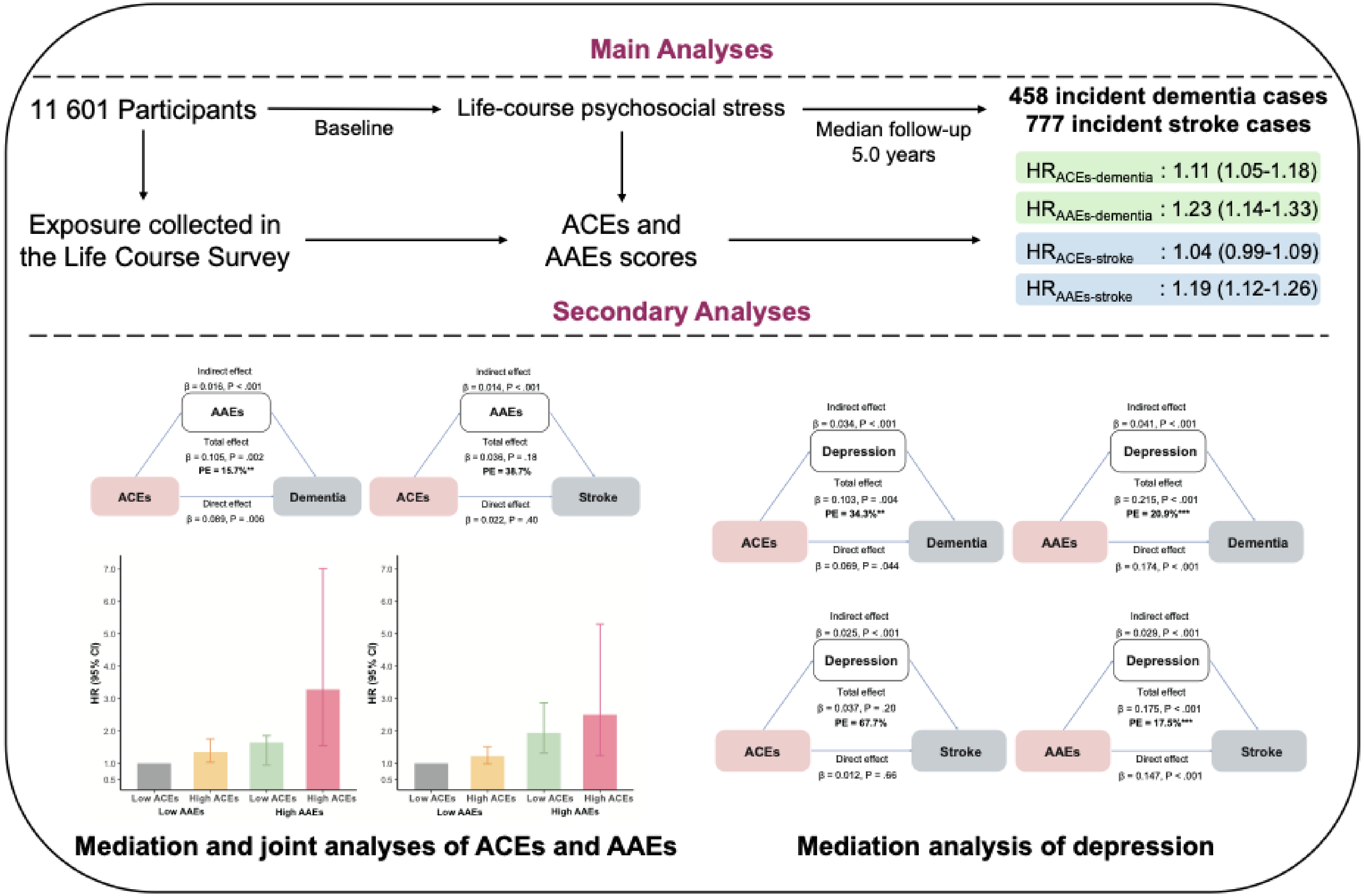

© 2026 Chen B et al. *JAMA Network Open*.

eMethods

Participants selection

This study primarily utilized data from the 2014 Life Course Survey and the main surveys of the CHARLS conducted in 2015, 2018, and 2020, with 2015 defined as the analytic baseline when exposure and covariate information were first available. After matching the 2014 life history survey data with the 2015 survey data on a 1:1 basis, we first excluded 1377 participants younger than 45 years or lacking age data. We then excluded 2958 individuals who were lost to follow-up during the period, followed by 4373 participants lacking ACEs or AAEs data. Finally, we excluded 461 participants with baseline dementia diagnoses and 325 with baseline stroke diagnoses. The final cohort comprised 11 601 participants (**eFigure 2** in Supplement 1).

Assessment of ACEs and AAEs

ACEs indicators included seven conventional ACEs (physical abuse, emotional neglect, household substance abuse, household mental illness, domestic violence, incarcerated household member, and parental separation or divorce), two expanded ACEs (unsafe neighborhood and bullying), and three newly identified ACEs previously reported in the literature (parental death, sibling death, and parental disability) [1]. Also, AAEs indicators included experiencing the death of the child, lifetime discrimination, ever being confined to bed, ever being hospitalized for a month or longer, and ever leaving a job due to health conditions [2]. All ACEs and AAEs information was collected in the 2014 Life Course Survey.

Latent class analysis

To identify potential subgroups of adversity exposure, latent class analysis (LCA) was conducted separately for adverse childhood experiences (ACEs, 12 items) and adverse adulthood experiences (AAEs, 5 items). Each item was treated as a binary indicator (exposed = 2, not exposed = 1). LCA was implemented using the poLCA package in R, with maximum likelihood estimation and random starting values to avoid local maxima [3]. Models with different numbers of latent classes were fitted (1–5 classes). The optimal number of classes was selected based on the lowest Bayesian information criterion (BIC). Item-response probabilities (posterior probabilities of endorsement for each item) were used to characterize each latent class. Based on these criteria, the optimal solution included four ACEs classes (low, lower-middle risk, upper-middle risk, and high) and three AAEs classes (low, medium, and high). Detailed class characteristics are provided in **eFigures 3-4**.

Definitions of dementia and stroke

Dementia was defined as the presence of both functional and cognitive impairments, or self-reported, doctor-diagnosed dementia or memory-related disorders [4-6]. The definition is

© 2026 Chen B et al. *JAMA Network Open*.

consistent with the criteria of the Diagnostic and Statistical Manual of Mental Disorders, 5th edition (DSM-5), and the International Classification of Diseases, 10th edition (ICD-10), for dementia diagnosis, and has been validated against clinical diagnoses in a nationwide multisite cross-sectional survey [4]. Memory-related diseases diagnosed by a physician, identified by the item "Have you been diagnosed with memory-related diseases?", used to identify participants without cognitive scores [7].

In CHARLS, functional impairment was assessed using the Katz Scale and defined as requiring care for one or more basic activities of daily living, including bathing, transferring in/out of bed, dressing, toileting, feeding, and continence [8,9]. Cognitive impairment was assessed using the Telephone Interview for Cognitive Status (TICS) questionnaire, which was administered through face-to-face interviews across all waves of the CHARLS [10]. The 9-item TICS, a modified version of the Mini-Mental State Examination, assesses four cognitive domains: time orientation, memory, visual structuring, and attention. The total score ranges from 0 to 31, with cognitive impairment defined as a score more than 1.5 standard deviations below the mean cognitive score of participants with the same educational level [11,12].

Stroke events were identified through participants' responses to the question: "Have you ever been diagnosed with a stroke by a doctor?", and events were confirmed by self-reported physician diagnoses.

Follow-up time was calculated from baseline (2015) to the date of incident dementia or stroke, death, or last observation, whichever occurred first.

Assessment of depression

Depressive symptoms were assessed at baseline (2015) using the 10-item Centre for Epidemiologic Studies Depression Scale (CES-D-10) [13]. Each item assessed respondents' feelings and behaviors over the past week, scored 0–3, yielding a total score of 0–30 (**eTable 3**). A dichotomous variable with a cut-off of 10 was used to classify participants as having depression (≥10) or not (<10) [14]. This approach has been widely validated in previous studies [15,16].

Covariates

Covariates were selected based on baseline interview data. These covariates included age (years), sex (male or female), education level (no formal education, primary, secondary school, above high school), marital status (married or single), smoking status (yes or no), drinking status (yes or no), sleep duration (≤6 hours or >6 hours), diabetes (yes or no), diabetes medication (yes or no), heart disease (yes or no), and cancer history (yes or no). We developed 3 Cox models: Model 1 was unadjusted for any covariates; Model 2 was adjusted for age, sex; Model 3 was fully adjusted for education, marital status, smoking status, drinking status, sleep, history of diabetes, diabetes medication, history of heart disease and cancer history based on Model 2.

© 2026 Chen B et al. *JAMA Network Open*.

In sensitivity analyses, additional covariates were included to evaluate the robustness of our findings. The socioeconomic status (SES; low, lower-middle, upper-middle, high), parental education (no formal education, primary, secondary school, above high school), and residential type (urban or rural) were initially considered. Furthermore, we additionally adjusted for biomarkers, including systolic blood pressure (SBP), diastolic blood pressure (DBP), body mass index (BMI), glucose, triglycerides, high-density lipoprotein (HDL), and C-reactive protein (CRP). Based on previous studies, SES was defined using two indicators: educational level and total household wealth [17]. Educational level was harmonized into three categories—primary (scored 0), secondary (scored 1), and tertiary (scored 2)—according to study-specific classifications. Total household wealth was calculated as the sum of all major assets, including residence, business, vehicles, and savings accounts, excluding any debts or loans. Wealth was ranked into quartiles, with scores ranging from 0 (lowest, quartile 1) to 3 (highest, quartile 4). The SES index was derived by summing the scores for education and wealth, and further categorized into four levels: low (score 0), lower-middle (scores 1–2), upper-middle (scores 3–4), and high (score 5). Parental education level was recorded as the highest level of education attained by either parent [18].

Sensitivity analyses

First, we conducted regression analyses for each individual component of AAEs and ACEs separately to examine their independent effects. Second, the main analysis was rerun after excluding samples with missing covariate data to assess the impact of missing data. Third, participants diagnosed with cancer history at baseline were excluded to examine the potential confounding effect of pre-existing disease. Fourth, dementia was redefined strictly based on cognitive impairment and functional impairment, excluding self-reported physician diagnosis, to improve diagnostic accuracy. Fifth, when incident dementia and stroke were the outcomes, the Fine-Gray sub-distribution hazard model was applied to account for the potential impact of mortality as a competing event. Sixth, additional adjustments were performed for SES, parental education, and residential type, with stratified analyses performed accordingly. Seventh, biomarkers including SBP, DBP, BMI, glucose, triglycerides, HDL, and CRP were further adjusted to examine physiological influences. Eighth, interaction terms between covariates were additionally included to explore potential interaction effects.

© 2026 Chen B et al. *JAMA Network Open*.

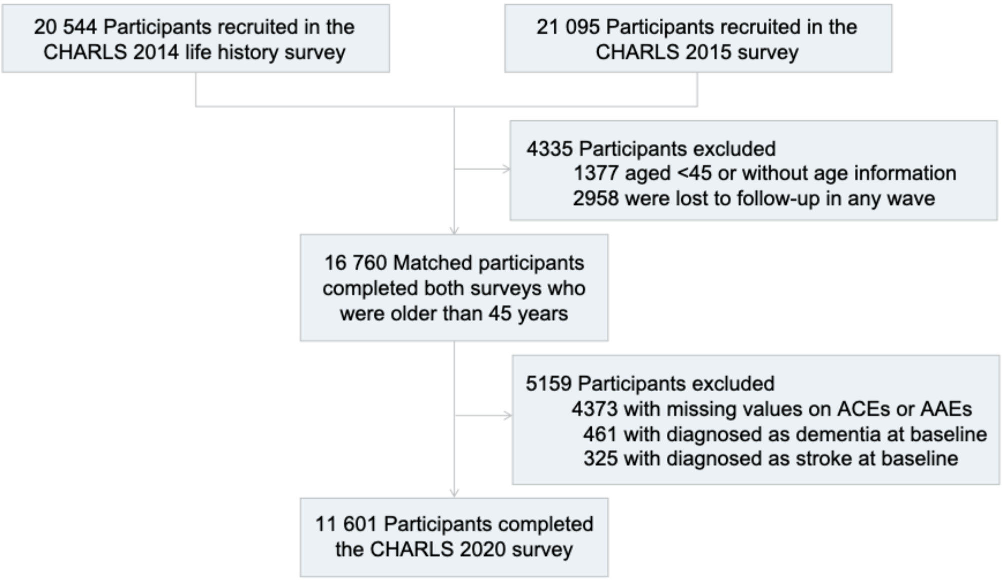

eFigure 2. Flowchart of the included population

AAEs indicates adverse adulthood experiences; ACEs, adverse childhood experiences; and CHARLS, China Health and Retirement Longitudinal Study.

© 2026 Chen B et al. *JAMA Network Open*.

eTable 1. Baseline characteristics of included and excluded participants

Characteristic	Overall [a] (N = 21 095)	Excluded [a] (N = 9494)	Included (N = 11 601)	P value [b]
Age, years, mean (SD)	59.09 (10.75)	58.97 (12.21)	59.18 (9.41)	<.001
Sex, n (%)				.34
Female	11 029 (52.3)	4997 (52.7)	6032 (52.0)	
Male	10 061 (47.7)	4492 (47.3)	5569 (48.0)	
Marital status, n (%)				<.001
Married	18 292 (86.7)	8037 (84.7)	10 255 (88.4)	
Single	2803 (13.3)	1457 (15.3)	1346 (11.6)	
Education, n (%)				<.001
Above high school	856 (4.1)	487 (5.1)	369 (3.2)	
Secondary school	5410 (25.7)	2163 (22.8)	3247 (28.0)	
Primary	9584 (45.5)	4416 (46.6)	5168 (44.5)	
No formal education	5225 (24.8)	2408 (25.4)	2817 (24.3)	
Drinking, n (%)				.06
No	11 239 (53.7)	5077 (54.4)	6162 (53.1)	
Yes	9693 (46.3)	4254 (45.6)	5439 (46.9)	
Smoking, n (%)				.29
No	11 908 (56.8)	5352 (57.2)	6556 (56.5)	
Yes	9043 (43.2)	3998 (42.8)	5045 (43.5)	
Sleep, n (%)				.16
≤6 hours	9890 (49.8%)	4158 (50.4%)	5732 (49.4%)	
>6 hours	9958 (50.2%)	4089 (49.6%)	5869 (50.6%)	
Diabetes, n (%)				.002
No	16 793 (90.2)	6272 (89.3)	10 521 (90.7)	
Yes	1831 (9.8)	751 (10.7)	1080 (9.3)	
Heart disease, n (%)				<.001
No	15 360 (82.3)	5686 (80.6)	9674 (83.4)	
Yes	3294 (17.7)	1367 (19.4)	1927 (16.6)	
Cancer history, n (%)				.10
No	18 358 (98.3)	6944 (98.1)	11 414 (98.4)	
Yes	324 (1.7)	137 (1.9)	187 (1.6)	
ACEs score, mean (SD)	1.70 (1.42)	1.70 (1.45)	1.70 (1.41)	.45
ACEs category, n (%)				.05
0	3244 (21.4)	788 (22.0)	2456 (21.2)	
1	4560 (30.0)	1101 (30.7)	3459 (29.8)	
2	3525 (23.2)	768 (21.4)	2757 (23.8)	
3	2151 (14.2)	507 (14.1)	1644 (14.2)	
≥4	1709 (11.3)	424 (11.8)	1285 (11.1)	
AAEs score, mean (SD)	0.69 (1.05)	0.76 (1.09)	0.64 (1.01)	<.001
AAEs category, n (%)				<.001

© 2026 Chen B et al. *JAMA Network Open*.

0	11 408 (61.4)	4048 (57.9)	7360 (63.4)
1	3718 (20.0)	1460 (20.9)	2258 (19.5)
2	1849 (9.9)	766 (11.0)	1083 (9.3)
3	1176 (6.3)	524 (7.5)	652 (5.6)
≥4	437 (2.4)	189 (2.7)	248 (2.1)

Abbreviations: AAEs, adverse adulthood experiences; ACEs, adverse childhood experiences; SD, Standard Deviation.

[a] Counts may not sum to column totals because of missing data.

[b] Continuous variables were compared using the Wilcoxon rank sum test; categorical variables were compared using Pearson's Chi-squared test.

© 2026 Chen B et al. *JAMA Network Open*.

eTable 2. Questionnaire items and responses of each ACEs and AAEs indicator

Categories	Domain	Questionnaire items
ACEs		
Conventional ACEs	Physical abuse	When you were growing up, did your female/male guardian ever hit you? (Responses were categorized as 0=rarely or never and 1=often or sometimes)
	Emotional neglect	How much love and affection did your female guardian give you while you were growing up? (Responses were categorized as 0=often or sometimes and 1=rarely or never)
		How much effort did your female guardian put into watching over you? (Responses were categorized as 0= a lot or some and 1=a litter or not at all)
	Household substance abuse	During the years you were growing up, did your female/male guardian ever have alcoholism or drug? (Responses were categorized as 0=no and 1=yes)
	Household mental illness	Did your female/male guardian have abnormality of mind when you were young? (Responses were categorized as 0=no and 1=yes)
		During the years you were growing up, had your female/male guardian often showed continued signs of sadness or depression? (Responses were categorized as 0=some or only a little of the childhood and 1=during all or most)
	Domestic violence	Have your father/mother ever beat up your mother/father? (Responses were categorized as 0=rarely or never and 1=often or sometimes)
	Incarcerated household member	During the years you were growing up, have your female/male guardian ever been arrested or sent to prison? (Responses were categorized as 0=no and 1=yes)
	Parental separation or divorce	Were your biological parents divorced (including long separation due to emotional problems) before you were 17 years? (Responses were categorized as 0=no and 1=yes)
Expanded ACEs	Unsafe neighborhood	Was it safe being out alone at night in the neighborhood where you lived as a child? (Responses were categorized as 0=very safe or somewhat safe and 1=not very safe or not safe at all)
	Bullying	When you were a child, how often were you picked on or bullied by kids in your neighborhood? (Responses were categorized as 0=not very often or never and 1=often or sometimes)

© 2026 Chen B et al. *JAMA Network Open*.

		When you were a child, how often were you picked on or bullied by kids in your school? (Responses were categorized as 0=not very often or never and 1=often or sometimes)
New ACEs	Parental death	Either of the parents was dead before participant was 17 years. (Responses were categorized as 0=no and 1=yes)
	Sibling death	Any of the siblings was dead before participant was 17 years. (Responses were categorized as 0=no and 1=yes)
	Parental disability	Did your female/male guardian have a long time being sick on bed when you were young? (Responses were categorized as 0=no and 1=yes)
		Did your female/male guardian have a serious deformity when you were young? (Responses were categorized as 0=no and 1=yes)
AAEs		
	Death of the child	Death of the participant's child (Responses were categorized as 0=no and 1=yes).
	Experiencing lifetime discrimination	After you were 16 years old, because of ill health, did you experience any of the following (denied promotions, assignment to a task with fewer responsibilities, working on tasks below your qualifications, harassment by your boss or colleagues, pay cuts, dismissed)? (Responses were categorized as 0=no and 1=yes).
	Ever being confined to bed	After you were 16 years old, because of a health condition, were you ever confined to bed or home for one month or more? (Responses were categorized as 0=no and 1=yes).
	Ever being hospitalized for a month or longer	After you were 16 years old, because of a health condition, were you ever hospitalized for a month or more? (Responses were categorized as 0=no and 1=yes).
	Ever leaving a job due to health conditions	After you were 16 years old, because of a health condition, did you leave your job for one month or more? (Responses were categorized as 0=no and 1=yes).

Abbreviation: ACEs, adverse childhood experiences; AAEs, adverse adulthood experiences.

© 2026 Chen B et al. *JAMA Network Open*.

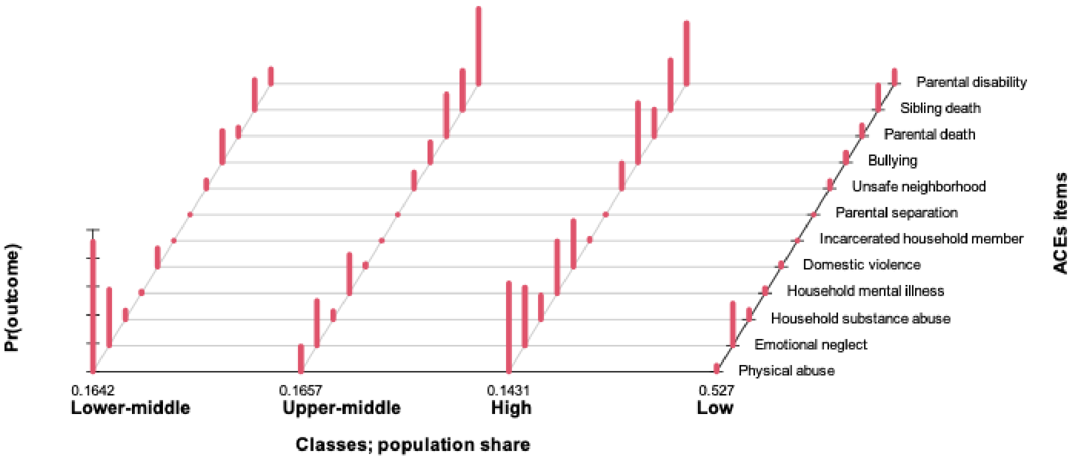

eFigure 3. Distribution of item-response probabilities across latent classes of ACEs

Four latent classes were identified: low , lower-middle, upper-middle, and high risk. ACEs indicates adverse childhood experiences; and Pr, Probability.

© 2026 Chen B et al. *JAMA Network Open*.

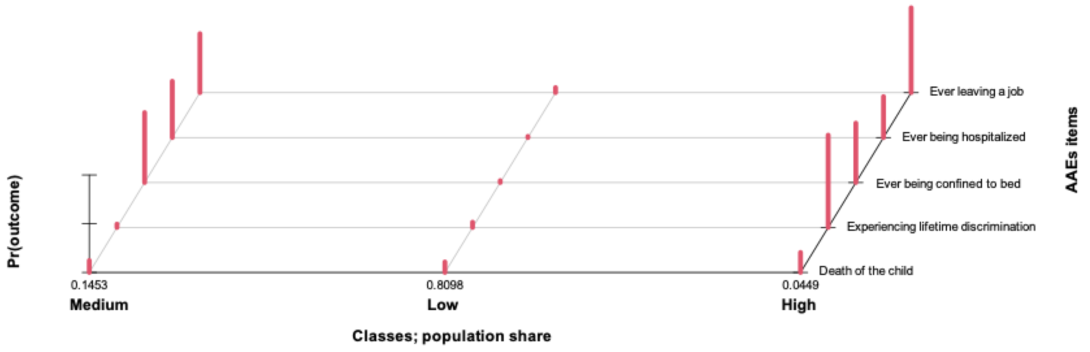

eFigure 4. Distribution of item-response probabilities across latent classes of AAEs

Three latent classes were identified: low, medium, and high risk. AAEs indicates adverse adulthood experiences; and Pr, Probability.

© 2026 Chen B et al. *JAMA Network Open*.

eTable 3. The items of the Center for Epidemiological Survey Depression Scale

Questionnaire items	Answers
I was bothered by things that don't usually bother me.	1. Rarely or none of the time (< 1 day)
	2. Some or a little of the time (1 − 2 days)
	3. Occasionally or a moderate amount of the time (3 − 4 days)
	4. Most or all of the time (5 − 7 days)
I had trouble keeping my mind on what I was doing.	1. Rarely or none of the time (< 1 day)
	2. Some or a little of the time (1 − 2 days)
	3. Occasionally or a moderate amount of the time (3 − 4 days)
	4. Most or all of the time (5 − 7 days)
I felt depressed.	1. Rarely or none of the time (< 1 day)
	2. Some or a little of the time (1 − 2 days)
	3. Occasionally or a moderate amount of the time (3 − 4 days)
	4. Most or all of the time (5 − 7 days)
I felt everything I did was an effort.	1. Rarely or none of the time (< 1 day)
	2. Some or a little of the time (1 − 2 days)
	3. Occasionally or a moderate amount of the time (3 − 4 days)
	4. Most or all of the time (5 − 7 days)
I felt hopeful about the future. [a]	1. Rarely or none of the time (< 1 day)
	2. Some or a little of the time (1 − 2 days)
	3. Occasionally or a moderate amount of the time (3 − 4 days)
	4. Most or all of the time (5 − 7 days)
I felt fearful.	1. Rarely or none of the time (< 1 day)
	2. Some or a little of the time (1 − 2 days)
	3. Occasionally or a moderate amount of the time (3 − 4 days)
	4. Most or all of the time (5 − 7 days)
My sleep was restless.	1. Rarely or none of the time (< 1 day)
	2. Some or a little of the time (1 − 2 days)
	3. Occasionally or a moderate amount of the time (3 − 4 days)
	4. Most or all of the time (5 − 7 days)
I was happy. [a]	1. Rarely or none of the time (< 1 day)
	2. Some or a little of the time (1 − 2 days)

© 2026 Chen B et al. *JAMA Network Open*.

	3. Occasionally or a moderate amount of the time (3 − 4 days)<br>4. Most or all of the time (5 − 7 days)
I felt lonely.	1. Rarely or none of the time (< 1 day)<br>2. Some or a little of the time (1 − 2 days)<br>3. Occasionally or a moderate amount of the time (3 − 4 days)<br>4. Most or all of the time (5 − 7 days)
I could not get “going”	1. Rarely or none of the time (< 1 day)<br>2. Some or a little of the time (1 − 2 days)<br>3. Occasionally or a moderate amount of the time (3 − 4 days)<br>4. Most or all of the time (5 − 7 days)

[a] The values for were reverse-coded.

eTable 4. Missing data proportions for covariates

Variable	Missing amount (n)	Missing ratio (%)
Diabetes medication	18	0.16
Smoking	20	0.17
Drinking	34	0.29
Sleep	443	3.82
Cancer history	1679	14.5
Heart disease	1692	14.6
Diabetes	1748	15.1
Sensitivity analyses		
Parental education	87	0.75
SES	174	1.50

Abbreviation: SES, socioeconomic status.

© 2026 Chen B et al. *JAMA Network Open*.

eTable 5. Summary of Cox proportional hazards assumption tests [a]

Variable	AAEs-dementia		ACEs-dementia		AAEs-stroke		ACEs-stroke	
	**Chisq**	**P value**	**Chisq**	**P value**	**Chisq**	**P value**	**Chisq**	**P value**
Main Predictor								
AAEs	1.569	.21	NA	NA	3.027	.08	NA	NA
ACEs	NA	NA	0.323	.57	NA	NA	1.368	.24
Covariates								
Age	0.004	.95	<0.001	.99	0.066	.80	0.063	.80
Sex	0.057	.81	0.060	.81	0.011	.92	0.008	.93
Education	1.479	.69	1.623	.65	5.050	.17	4.972	.17
Marital status	0.395	.53	0.438	.51	0.125	.72	0.121	.73
Smoking	0.024	.88	0.022	.88	0.185	.67	0.175	.68
Drinking	0.197	.66	0.227	.63	0.015	.90	0.006	.94
Sleep	0.227	.63	0.215	.64	0.417	.52	0.398	.53
Diabetes medication	0.766	.38	0.713	.40	0.057	.81	0.040	.84
Diabetes	0.756	.38	0.640	.42	0.030	.86	0.019	.89
Heart disease	0.227	.63	0.252	.62	0.008	.93	0.010	.92
Cancer history	2.233	.14	2.186	.14	0.520	.47	0.524	.47
GLOBAL test	7.689	.90	6.788	.94	9.722	.78	8.191	.88

Abbreviation: AAEs, adverse adulthood experiences; ACEs, adverse childhood experiences; NA, not applicable.

[a] The analysis was performed in Model 3 (adjusted with age, sex, education, marital status, smoking status, drinking status, sleep, diabetes, diabetes medication, heart disease and cancer history).

© 2026 Chen B et al. *JAMA Network Open*.

eTable 6. GVIF-based multicollinearity diagnostics for covariates included in Model 3 [a]

Variable	AAEs-dementia		ACEs-dementia		AAEs-stroke		ACEs-stroke	
	GVIF	$GVIF^{1/(2 \cdot Df)}$	GVIF	$GVIF^{1/(2 \cdot Df)}$	GVIF	$GVIF^{1/(2 \cdot Df)}$	GVIF	$GVIF^{1/(2 \cdot Df)}$
Age	1.30	1.14	1.30	1.14	1.26	1.12	1.02	1.01
Sex	2.58	1.61	2.56	1.60	2.69	1.64	1.26	1.12
Education	1.32	1.05	1.31	1.05	1.30	1.04	2.69	1.64
Marital status	1.21	1.10	1.21	1.10	1.16	1.08	1.30	1.04
Smoking	2.12	1.45	2.11	1.45	2.21	1.49	1.16	1.08
Drinking	1.36	1.17	1.37	1.17	1.39	1.18	2.21	1.49
Sleep	1.02	1.01	1.02	1.01	1.02	1.01	1.40	1.18
Diabetes medication	2.10	1.45	2.12	1.45	2.00	1.42	1.02	1.01
Diabetes	2.13	1.46	2.15	1.47	2.04	1.43	2.01	1.42
Heart	1.07	1.03	1.07	1.03	1.08	1.04	2.05	1.43
Cancer history	1.02	1.01	1.01	1.01	1.01	1.00	1.07	1.03

Abbreviation: AAEs, adverse adulthood experiences; ACEs, adverse childhood experiences; Df, degrees of freedom; GVIF, generalized variance inflation factor.

[a] The analysis was performed in Model 3 (adjusted with age, sex, education, marital status, smoking status, drinking status, sleep, diabetes, diabetes medication, heart disease and cancer history). GVIF indicates generalized variance inflation factor, and $GVIF^{1/(2 \cdot Df)}$ represents the adjusted GVIF accounting for degrees of freedom (Df). A $GVIF^{1/(2 \cdot Df)} < 2$ generally indicates no concerning multicollinearity.

© 2026 Chen B et al. *JAMA Network Open*.

eTable 7. Interaction analyses among covariates included in Model 3 [a] for associations of ACEs and AAEs with incident dementia

Variable1	Variable2	P interaction [b]
Age	Marital status	.006
Age	Diabetes medication	.02
Diabetes medication	Diabetes	.03
Age	Heart disease	.09
Marital status	Cancer history	.09
Age	Education	.12
Heart disease	Cancer history	.14
Age	Sex	.16
Diabetes medication	Cancer history	.16
Marital status	Diabetes	.18
Drinking	Diabetes medication	.21
Sleep	Cancer history	.22
Sex	Cancer history	.23
Education	Heart disease	.25
Education	Diabetes	.26
Drinking	Heart disease	.28
Sex	Education	.32
Sex	Marital status	.32
Smoking	Heart disease	.33
Diabetes	Cancer history	.35
Drinking	Diabetes	.38
Age	Sleep	.41
Diabetes medication	Heart disease	.42
Education	Drinking	.43
Age	Diabetes	.44
Sleep	Diabetes medication	.48
Education	Smoking	.50
Smoking	Cancer history	.51
Sleep	Heart disease	.52
Marital status	Sleep	.54
Marital status	Drinking	.56
Marital status	Diabetes medication	.57
Marital status	Smoking	.58
Smoking	Diabetes	.60
Sex	Diabetes medication	.63
Smoking	Sleep	.64
Education	Sleep	.65
Smoking	Diabetes medication	.66
Diabetes	Heart disease	.71

© 2026 Chen B et al. *JAMA Network Open*.

Drinking	Cancer history	.73
Sex	Smoking	.75
Sex	Sleep	.75
Sleep	Diabetes	.75
Smoking	Drinking	.75
Sex	Diabetes	.75
Education	Marital status	.77
Age	Cancer history	.78
Sex	Heart disease	.86
Age	Smoking	.88
Marital status	Heart disease	.91
Education	Diabetes medication	.93
Age	Drinking	.94
Sex	Drinking	.98
Education	Cancer history	.99
Drinking	Sleep	.99

Abbreviation: AAEs, adverse adulthood experiences; ACEs, adverse childhood experiences.

[a] Model 3 included age, sex, education, marital status, smoking status, drinking status, sleep, diabetes, diabetes medication, heart and cancer history.

[b] P value for interaction was derived by including a multiplicative interaction term in the Cox proportional hazards model.

eTable 8. Interaction analyses among covariates included in Model 3 [a] for associations of ACEs and AAEs with incident stroke

Variable1	Variable2	P interaction [b]
Age	Marital status	<.001
Sex	Heart disease	.001
Age	Sex	.004
Age	Diabetes	.005
Age	Smoking	.005
Smoking	Sleep	.006
Smoking	Diabetes medication	.02
Diabetes medication	Diabetes	.02
Age	Heart disease	.03
Drinking	Sleep	.03
Sex	Sleep	.03
Sex	Diabetes medication	.04
Diabetes	Heart disease	<.05 [c]
Age	Diabetes medication	<.05 [d]
Education	Sleep	.05 [e]
Diabetes medication	Heart disease	.05 [f]
Drinking	Heart disease	.09
Sex	Diabetes	.11
Drinking	Diabetes medication	.11
Smoking	Heart disease	.12
Education	Marital status	.16
Smoking	Diabetes	.17
Smoking	Drinking	.19
Sleep	Diabetes medication	.20
Diabetes	Cancer history	.20
Age	Sleep	.23
Diabetes medication	Cancer history	.24
Education	Diabetes	.29
Education	Smoking	.29
Education	Drinking	.29
Smoking	Cancer history	.33
Sex	Education	.36
Age	Cancer history	.36
Sex	Marital status	.40
Marital status	Diabetes medication	.50
Age	Drinking	.53
Sleep	Cancer history	.54
Marital status	Smoking	.54
Education	Heart disease	.55

© 2026 Chen B et al. *JAMA Network Open*.

Sleep	Diabetes	.56
Education	Diabetes medication	.57
Drinking	Diabetes	.58
Education	Cancer history	.63
Sex	Smoking	.68
Sex	Drinking	.70
Marital status	Heart disease	.71
Marital status	Sleep	.72
Drinking	Cancer history	.76
Marital status	Diabetes	.84
Marital status	Drinking	.87
Marital status	Cancer history	.87
Sex	Cancer history	.89
Heart disease	Cancer history	.89
Sleep	Heart disease	.93
Age	Education	.98

Abbreviation: AAEs, adverse adulthood experiences; ACEs, adverse childhood experiences.

[a] Model 3 included age, sex, education, marital status, smoking status, drinking status, sleep, diabetes, diabetes medication, heart and cancer history.

[b] P value for interaction was derived by including a multiplicative interaction term in the Cox proportional hazards model.

[c] The raw P value is .0466.

[d] The raw P value is .0499.

[e] The raw P value is .0510.

[f] The raw P value is .0537.

© 2026 Chen B et al. *JAMA Network Open*.

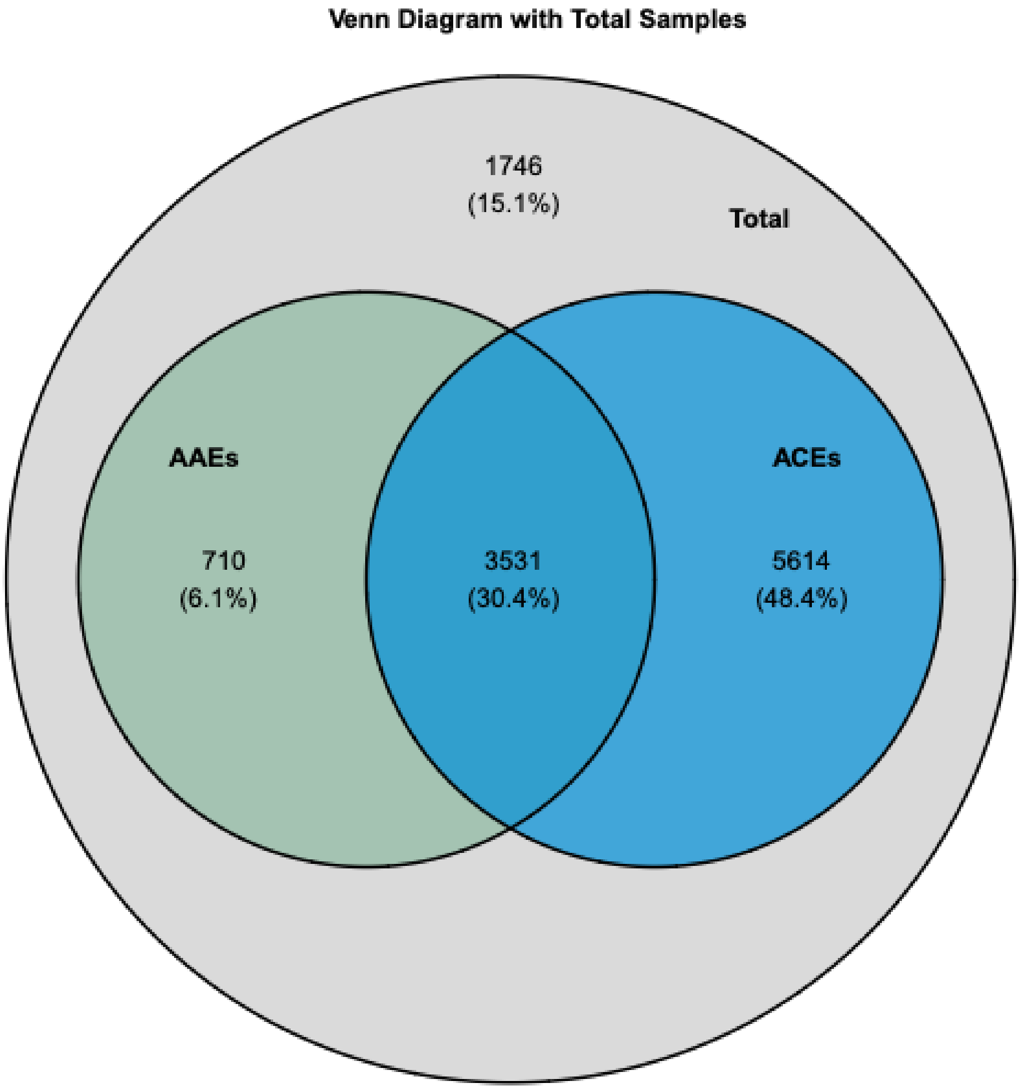

eFigure 5. Venn diagram of the study population by exposure status

The green area represents participants with AAEs, the blue area represents those with ACEs, and the grey area represents individuals without ACEs or AAEs. Numbers and percentages indicate the distribution of participants in each subgroup. AAEs indicates adverse adulthood experiences; and ACEs, adverse childhood experiences.

© 2026 Chen B et al. *JAMA Network Open*.

eTable 9. Baseline characteristics of study participants with 95% CI for OR (categorical variables) and mean differences (continuous variables) [a]

Characteristic	Dementia	P value	Stroke	P value
Age, mean differences (95% CI)	6.59 (5.69–7.48)	<.001	3.50 (2.86–4.15)	<.001
Sex, OR (95% CI)				
Female	Reference	NA	Reference	NA
Male	0.75 (0.62–0.91)	.003	0.98 (0.85–1.14)	.82
Marital status, OR (95% CI)				
Married	Reference	NA	Reference	NA
Single	1.95 (1.53–2.46)	<.001	1.35 (1.10–1.66)	.004
Education, OR (95% CI)				
Above high school	Reference	NA	Reference	NA
Secondary school	0.67 (0.39–1.25)	.18	0.86 (0.57–1.34)	.47
Primary	1.07 (0.64–1.94)	.81	0.93 (0.63–1.44)	.73
No formal education	1.44 (0.85–2.62)	.20	1.08 (0.72–1.68)	.72
Drinking, OR (95% CI)				
No	Reference	NA	Reference	NA
Yes	0.94 (0.78–1.13)	.52	0.94 (0.82–1.09)	.44
Smoking, OR (95% CI)				
No	Reference	NA	Reference	NA
Yes	0.83 (0.68–1.00)	.05	1.09 (0.94–1.27)	.23
Sleep, OR (95% CI)				
≤6 hours	Reference	NA	Reference	NA
>6 hours	0.78 (0.64–0.94)	.008	0.82 (0.71–0.95)	.009
Diabetes, OR (95% CI)				
No	Reference	NA	Reference	NA
Yes	1.94 (1.49–2.50)	<.001	2.13 (1.74–2.59)	<.001
Heart disease, OR (95% CI)				
No	Reference	NA	Reference	NA
Yes	2.38 (1.94–2.91)	<.001	2.26 (1.92–2.66)	<.001
Cancer history, OR (95% CI)				
No	Reference	NA	Reference	NA
Yes	1.84 (1.00–3.13)	.04	0.78 (0.39–1.41)	.46
ACEs score, mean differences (95% CI)	0.28 (0.14–0.43)	<.001	0.11 (0.01–0.22)	.04
ACEs category, OR (95% CI)				
0	Reference	NA	Reference	NA
1	1.28 (0.96–1.71)	.10	1.09 (0.88–1.35)	.41
2	1.29 (0.95–1.75)	.10	1.00 (0.80–1.26)	.98
3	1.52 (1.09–2.11)	.01	1.18 (0.92–1.51)	.19
≥4	1.91 (1.37–2.66)	<.001	1.39 (1.07–1.79)	.01
AAEs score, mean differences (95% CI)	0.38 (0.27–0.49)	<.001	0.29 (0.20–0.37)	<.001
AAEs category, OR (95% CI)				

© 2026 Chen B et al. *JAMA Network Open*.

0	Reference	NA	Reference	NA
1	2.03 (1.62–2.54)	<.001	1.37 (1.13–1.64)	.001
2	2.12 (1.58–2.82)	<.001	1.68 (1.33–2.11)	<.001
3	2.31 (1.61–3.22)	<.001	1.87 (1.41–2.45)	<.001
≥4	3.02 (1.82–4.75)	<.001	2.69 (1.82–3.86)	<.001

Abbreviations: AAEs, adverse adulthood experiences; ACEs, adverse childhood experiences; CI, confidence interval; NA, not applicable; OR, odds ratio.

[a] OR and CI for categorical variables were estimated using logistic regression models. Mean differences and corresponding 95% CI for continuous variables were calculated using two-sample t tests.

© 2026 Chen B et al. *JAMA Network Open*.

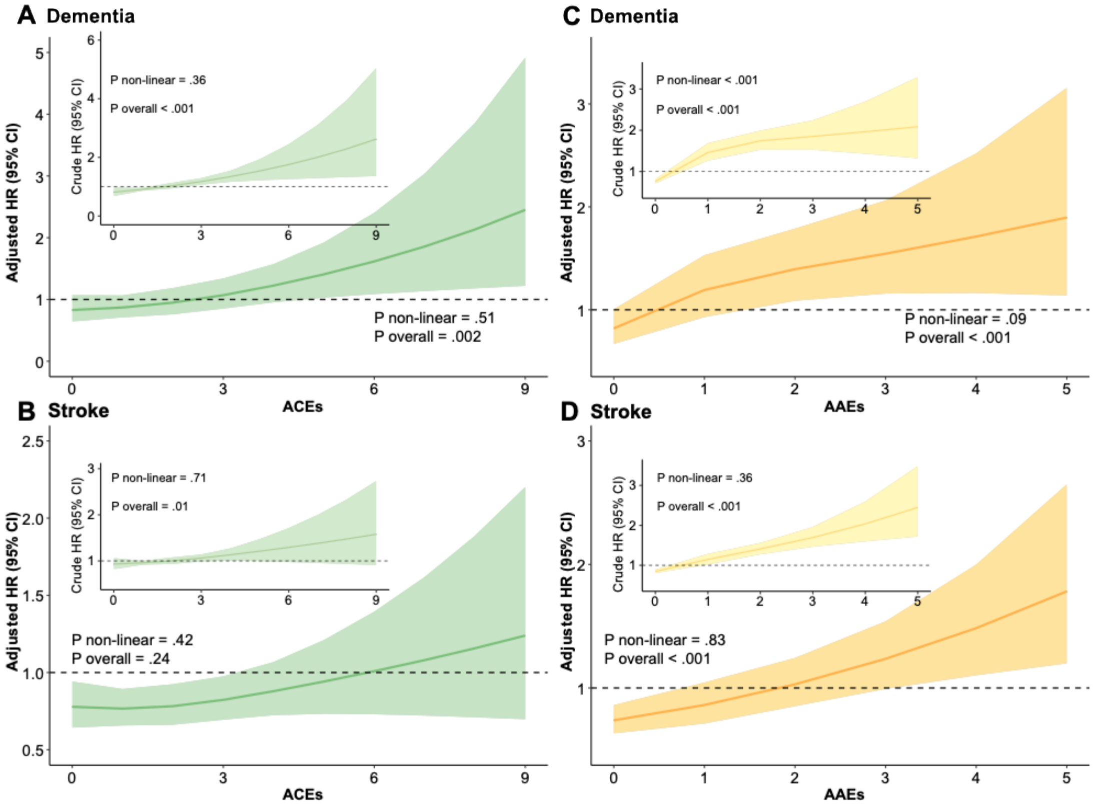

eFigure 6. RCS analyses of the associations of ACEs (A-B) and AAEs (C-D) with the risk of dementia and stroke

The main image was adjusted for the Model 3 (adjusted with age, sex, education, marital status, smoking status, drinking status, sleep, diabetes, diabetes medication, heart disease and cancer history), while the thumbnail image was not adjusted for any covariates. AAEs indicates adverse adulthood experiences; ACEs, adverse childhood experiences; CI, confidence interval; HR, hazard ratio; and RCS, restricted cubic spline.

© 2026 Chen B et al. *JAMA Network Open*.

eTable 10. Associations of latent ACEs and AAEs classes with incident dementia

	N	HR (95% CI) [a]	P value
ACEs latent classes [b]			
Low	6764	Reference	NA
Lower-middle	2356	1.03 (0.80-1.33)	.81
Upper-middle	1195	1.18 (0.89-1.58)	.26
High	1286	1.60 (1.23-2.08)	<.001
AAEs latent classes [b]			
Low	9444	Reference	NA
Medium	1570	1.53 (1.22-1.93)	<.001
High	587	1.51 (1.07-2.15)	.02

Abbreviation: AAEs, adverse adulthood experiences; ACEs, adverse childhood experiences; CI, confidence interval; HR, hazard ratio; NA, not applicable.

[a] The analysis was performed in Model 3 (adjusted with age, sex, education, marital status, smoking status, drinking status, sleep, diabetes, diabetes medication, heart disease and cancer history).

[b] ACEs and AAEs were categorized using latent class analysis based on the response probabilities of all individual indicators (12 ACEs items and 5 AAEs items). Four latent ACEs classes and three latent AAEs classes were identified, representing varying levels of adversity exposure.

© 2026 Chen B et al. *JAMA Network Open*.

eTable 11. Associations of latent ACEs and AAEs classes with incident stroke

	N	HR (95% CI) [a]	P value
ACEs latent classes [b]			
Low	6764	Reference	NA
Lower-middle	2356	1.13 (0.95-1.36)	.17
Upper-middle	1195	0.93 (0.73-1.19)	.56
High	1286	1.33 (1.08-1.65)	.008
AAEs latent classes [b]			
Low	9444	Reference	NA
Medium	1570	1.47 (1.23-1.76)	<.001
High	587	1.45 (1.10-1.92)	.008

Abbreviation: AAEs, adverse adulthood experiences; ACEs, adverse childhood experiences; CI, confidence interval; HR, hazard ratio; NA, not applicable.

[a] The analysis was performed in Model 3 (adjusted with age, sex, education, marital status, smoking status, drinking status, sleep, diabetes, diabetes medication, heart disease and cancer history).

[b] ACEs and AAEs were categorized using latent class analysis based on the response probabilities of all individual indicators (12 ACEs items and 5 AAEs items). Four latent ACEs classes and three latent AAEs classes were identified, representing varying levels of adversity exposure.

© 2026 Chen B et al. *JAMA Network Open*.

eTable 12. Associations of ACEs-binary and AAEs-binary groups with incident dementia

	N	HR (95% CI) [a]	P value
ACEs-binary groups			
0-3	10 316	Reference	NA
≥4	1285	1.40 (1.09-1.81)	.009
AAEs-binary groups			
0-3	11 353	Reference	NA
≥4	248	1.91 (1.21-2.99)	.005

Abbreviation: AAEs, adverse adulthood experiences; ACEs, adverse childhood experiences; CI, confidence interval; HR, hazard ratio; NA, not applicable.

[a] The analysis was performed in Model 3 (adjusted with age, sex, education, marital status, smoking status, drinking status, sleep, diabetes, diabetes medication, heart disease and cancer history).

© 2026 Chen B et al. *JAMA Network Open*.

eTable 13. Associations of ACEs-binary and AAEs-binary groups with incident stroke

	N	HR (95% CI) [a]	P value
ACEs-binary groups			
0-3	10 316	Reference	NA
≥4	1285	1.24 (1.01-1.52)	.04
AAEs-binary groups			
0-3	11 353	Reference	NA
≥4	248	1.99 (1.41-2.82)	<.001

Abbreviation: AAEs, adverse adulthood experiences; ACEs, adverse childhood experiences; CI, confidence interval; HR, hazard ratio; NA, not applicable.

[a] The analysis was performed in Model 3 (adjusted with age, sex, education, marital status, smoking status, drinking status, sleep, diabetes, diabetes medication, heart disease and cancer history).

© 2026 Chen B et al. *JAMA Network Open*.

eTable 14. Joint analyses of ACEs and AAEs with incident dementia and stroke

	N	Dementia HR (95% CI) [a]	P value	Stroke HR (95% CI) [a]	P value
ACEs & AAEs joint groups					
Low ACEs & Low AAEs	10 117	Reference	NA	Reference	NA
High ACEs & Low AAEs	1236	1.34 (1.03-1.75)	.03	1.22 (0.98-1.51)	.07
Low ACEs & High AAEs	199	1.64 (0.94-1.85)	.08	1.94 (1.31-2.87)	<.001
High ACEs & High AAEs	49	3.28 (1.54-7.02)	.002	2.50 (1.24-5.30)	.01
P interaction [b]			.42		.89

Abbreviation: AAEs, adverse adulthood experiences; ACEs, adverse childhood experiences; CI, confidence interval; HR, hazard ratio; NA, not applicable.

[a] The analysis was performed in Model 3 (adjusted with age, sex, education, marital status, smoking status, drinking status, sleep, diabetes, diabetes medication, heart disease and cancer history).

[b] P interaction was derived from the Wald test of the ACEs×AAEs interaction coefficient in the Cox proportional hazards model.

© 2026 Chen B et al. *JAMA Network Open*.

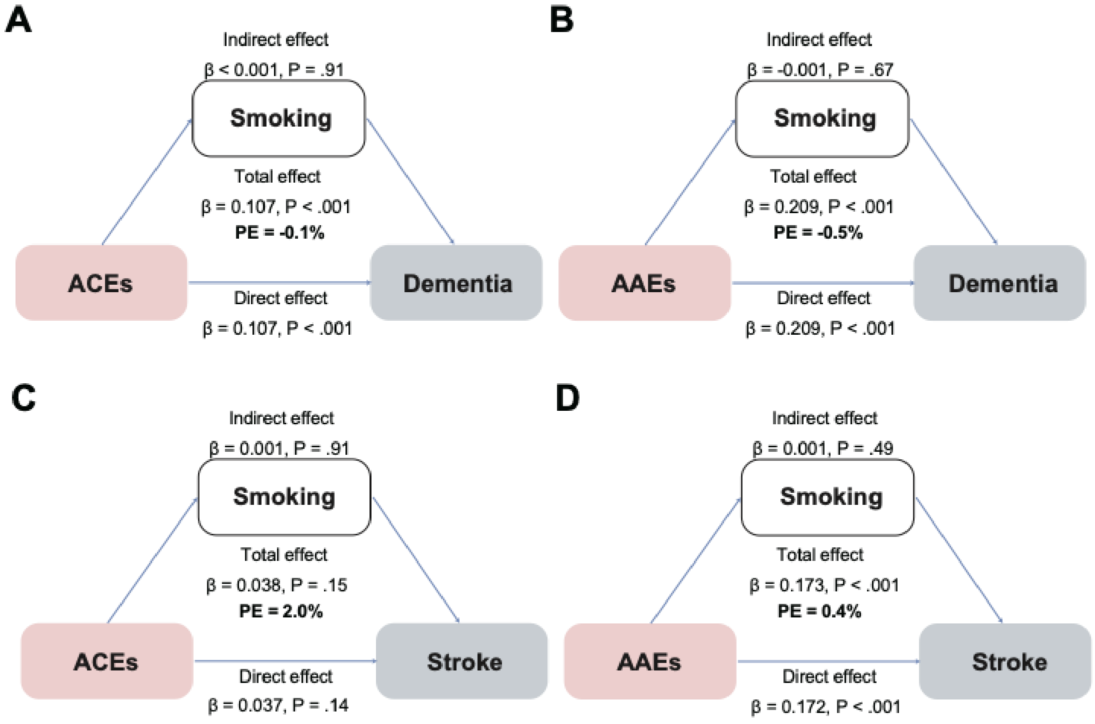

eFigure 7. Mediation analysis of smoking on associations of ACEs and AAEs with incident dementia and stroke

The analysis was adjusted with age, sex, education, marital status, drinking status, sleep, diabetes, diabetes medication, heart disease and cancer history. AAEs indicates adverse adulthood experiences; ACEs, adverse childhood experiences; and PE, proportion of mediation.

© 2026 Chen B et al. *JAMA Network Open*.

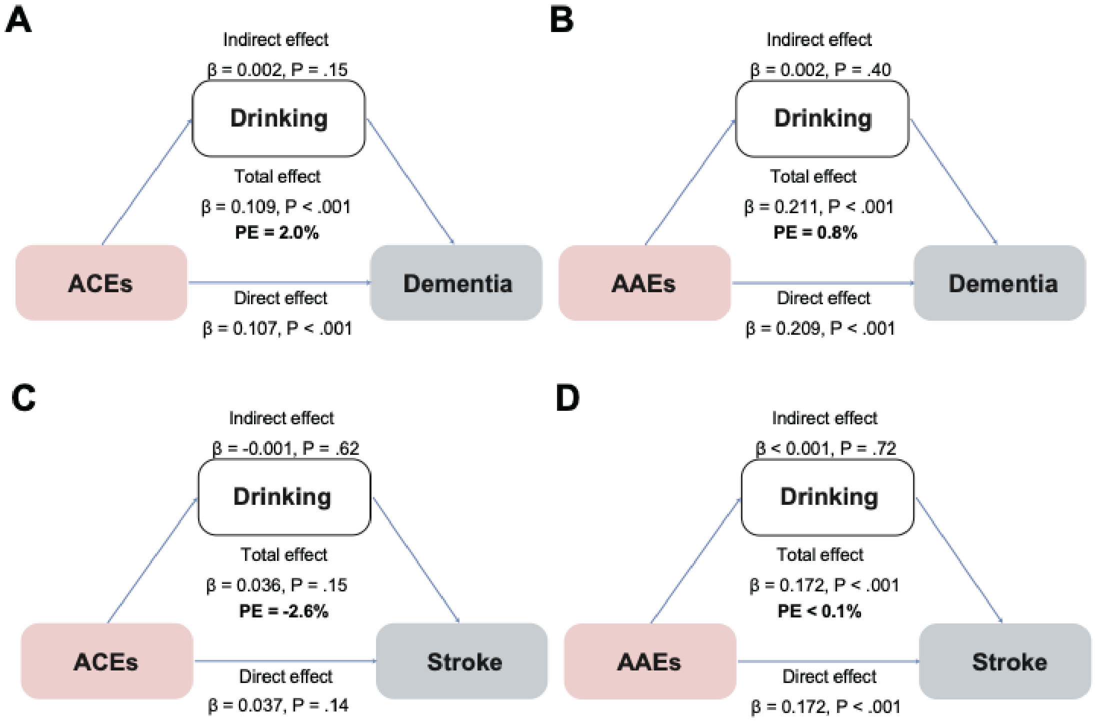

eFigure 8. Mediation analysis of drinking on associations of ACEs and AAEs with incident dementia and stroke

The analysis was adjusted with age, sex, education, marital status, smoking status, sleep, diabetes, diabetes medication, heart disease and cancer history. AAEs indicates adverse adulthood experiences; ACEs, adverse childhood experiences; and PE, proportion of mediation.

© 2026 Chen B et al. *JAMA Network Open*.

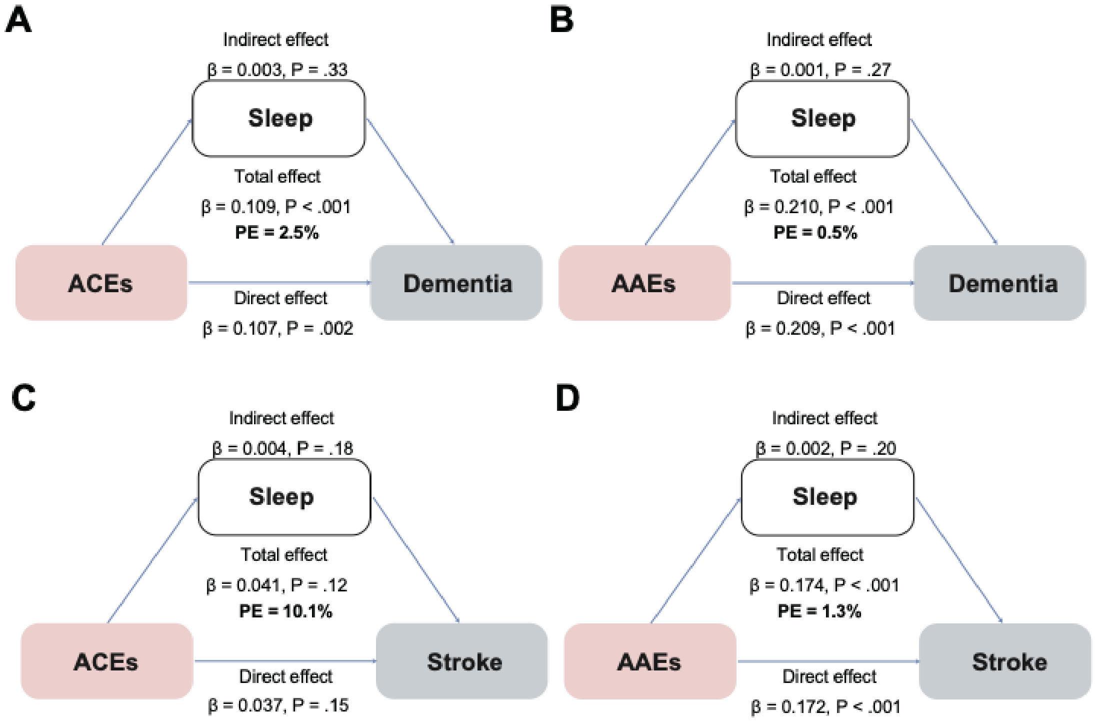

eFigure 9. Mediation analysis of sleep on associations of ACEs and AAEs with incident dementia and stroke

The analysis was adjusted with age, sex, education, marital status, smoking status, drinking status, diabetes, diabetes medication, heart disease and cancer history. AAEs indicates adverse adulthood experiences; ACEs, adverse childhood experiences; and PE, proportion of mediation.

© 2026 Chen B et al. *JAMA Network Open*.

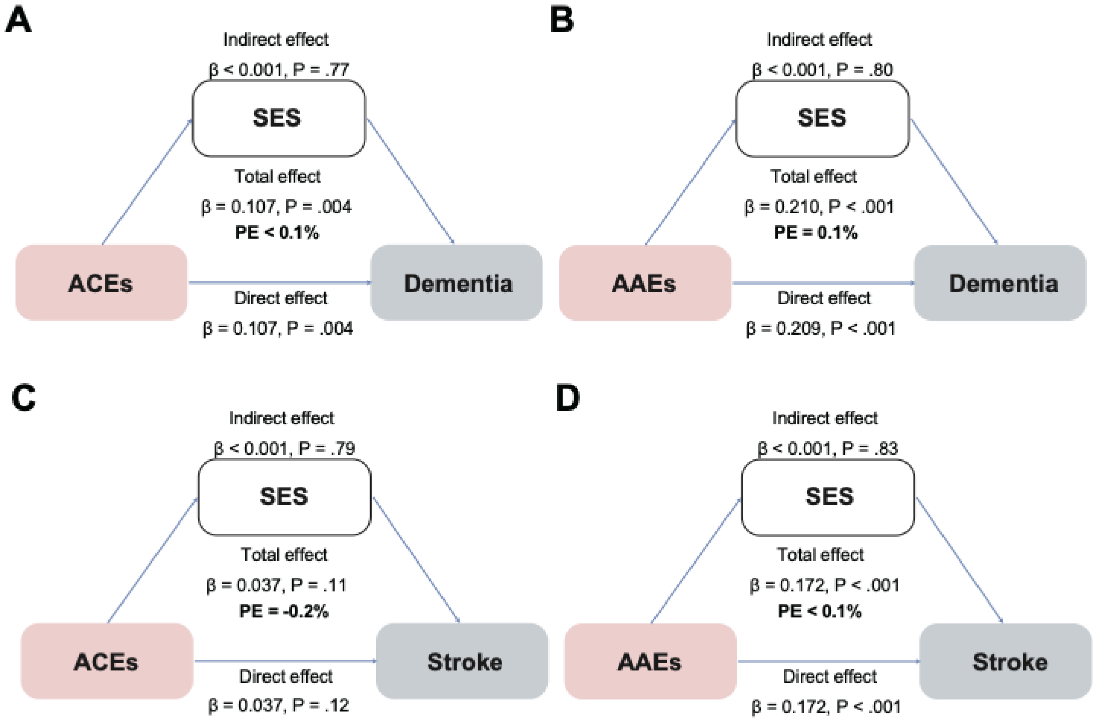

eFigure 10. Mediation analysis of SES on associations of ACEs and AAEs with incident dementia and stroke

The analysis was adjusted with age, sex, education, marital status, smoking status, drinking status, sleep, diabetes, diabetes medication, heart disease and cancer history. For ease of model interpretation, SES was dichotomized into low (SES score = 0) and high (SES score ≥ 1). AAEs indicates adverse adulthood experiences; ACEs, adverse childhood experiences; PE, proportion of mediation; and SES, socioeconomic status.

© 2026 Chen B et al. *JAMA Network Open*.

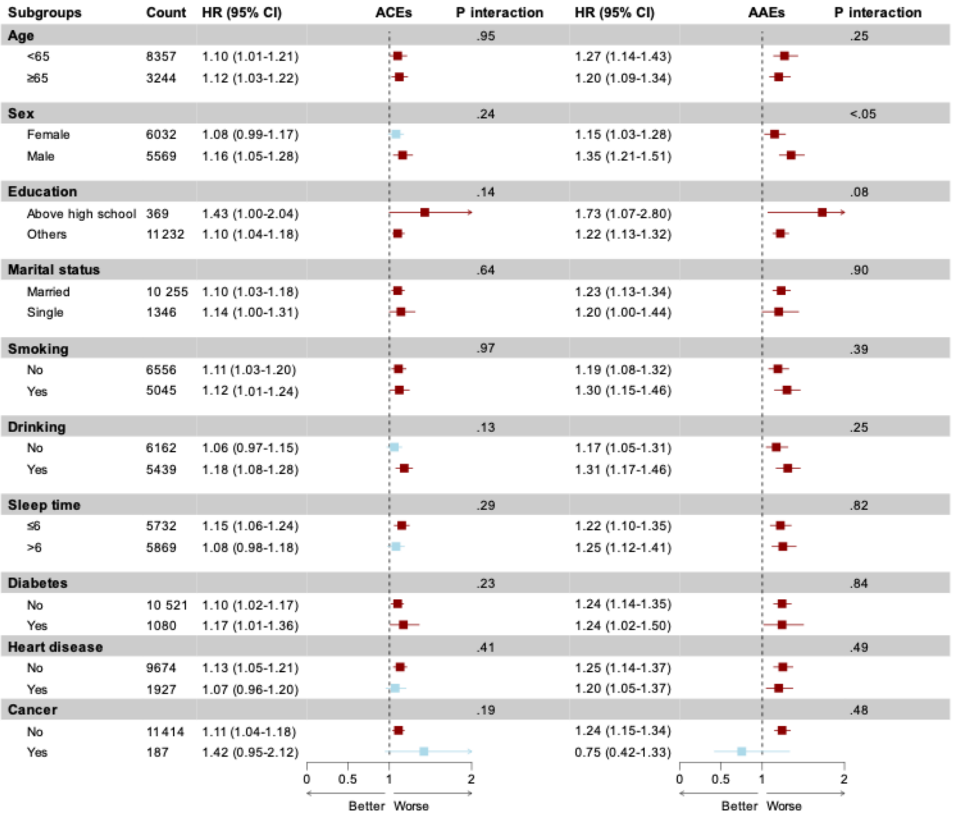

eFigure 11. Subgroup analysis of the associations of ACEs and AAEs with incident dementia

In the forest plot, red squares indicate statistically significant associations, whereas blue squares indicate non-significant associations. The analysis was performed in Model 3 (adjusted with age, sex, education, marital status, smoking status, drinking status, sleep, diabetes, diabetes medication, heart disease and cancer history). AAEs indicates adverse adulthood experiences; ACEs, adverse childhood experiences; CI, confidence interval; and HR, hazard ratio.

© 2026 Chen B et al. *JAMA Network Open*.

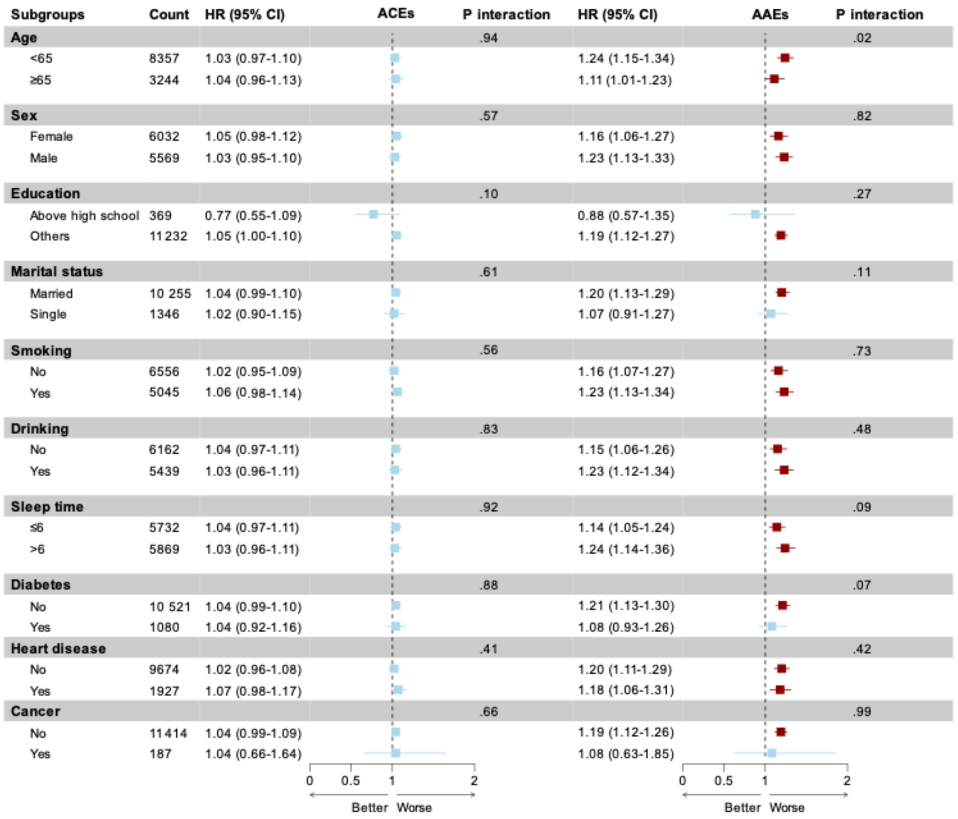

eFigure 12. Subgroup analysis of the associations of ACEs and AAEs with incident stroke

In the forest plot, red squares indicate statistically significant associations, whereas blue squares indicate non-significant associations. The analysis was performed in Model 3 (adjusted with age, sex, education, marital status, smoking status, drinking status, sleep, diabetes, diabetes medication, heart disease and cancer history). AAEs indicates adverse adulthood experiences; ACEs, adverse childhood experiences; CI, confidence interval; and HR, hazard ratio.

© 2026 Chen B et al. *JAMA Network Open*.

eTable 15. Subgroup analysis of the associations of ACEs and AAEs with incident dementia

Subgroups	Count	ACEs HR (95% CI) [a]	P interaction [b]	AAEs HR (95% CI) [a]	P interaction [b]
Age					
<65	8357	1.10 (1.01-1.21)	.95	1.27 (1.14-1.43)	.25
≥65	3244	1.12 (1.03-1.22)		1.20 (1.09-1.34)	
Sex					
Female	6032	1.08 (0.99-1.17)	.24	1.15 (1.03-1.28)	<.05 [c]
Male	5569	1.16 (1.05-1.28)		1.35 (1.21-1.51)	
Education					
Above high school	369	1.43 (1.00-2.04)	.14	1.73 (1.07-2.80)	.08
Others [d]	11 232	1.10 (1.04-1.18)		1.22 (1.13-1.32)	
Marital status					
Married	10 255	1.10 (1.03-1.18)	.64	1.23 (1.13-1.34)	.90
Single	1346	1.14 (1.00-1.31)		1.20 (1.00-1.44)	
Smoking					
No	6556	1.11 (1.03-1.20)	.97	1.19 (1.08-1.32)	.39
Yes	5045	1.12 (1.01-1.24)		1.30 (1.15-1.46)	
Drinking					
No	6162	1.06 (0.97-1.15)	.13	1.17 (1.05-1.31)	.25
Yes	5439	1.18 (1.08-1.28)		1.31 (1.17-1.46)	
Sleep time					
≤6	5732	1.15 (1.06-1.24)	.29	1.22 (1.10-1.35)	.82
>6	5869	1.08 (0.98-1.18)		1.25 (1.12-1.41)	
Diabetes					
No	10 521	1.10 (1.02-1.17)	.23	1.24 (1.14-1.35)	.84
Yes	1080	1.17 (1.01-1.36)		1.24 (1.02-1.50)	
Heart disease					
No	9674	1.13 (1.05-1.21)	.41	1.25 (1.14-1.37)	.49
Yes	1927	1.07 (0.96-1.20)		1.20 (1.05-1.37)	
Cancer history					
No	11 414	1.11 (1.04-1.18)	.19	1.24 (1.15-1.34)	.48
Yes	187	1.42 (0.95-2.12)		0.75 (0.42-1.33)	

Abbreviation: AAEs, adverse adulthood experiences; ACEs, adverse childhood experiences; CI, confidence interval; HR, hazard ratio.

[a] The analysis was performed in Model 3 (adjusted with age, sex, education, marital status, smoking status, drinking status, sleep, diabetes, diabetes medication, heart disease and cancer history).

[b] P value for interaction was derived by including a multiplicative interaction term in the Cox proportional hazards model.

[c] The raw P value is .0497.

[d] This category includes individuals with no formal education, primary school, or secondary school education levels.

© 2026 Chen B et al. *JAMA Network Open*.

eTable 16. Subgroup analysis of the associations of ACEs and AAEs with incident stroke

Subgroups	Count	ACEs HR (95% CI) [a]	P interaction [b]	AAEs HR (95% CI) [a]	P interaction [b]
Age					
<65	8357	1.03 (0.97-1.10)	.94	1.24 (1.15-1.34)	.02
≥65	3244	1.04 (0.96-1.13)		1.11 (1.01-1.23)	
Sex					
Female	6032	1.05 (0.98-1.12)	.57	1.16 (1.06-1.27)	.82
Male	5569	1.03 (0.95-1.10)		1.23 (1.13-1.33)	
Education					
Above high school	369	0.77 (0.55-1.09)	.10	0.88 (0.57-1.35)	.27
Others [c]	11 232	1.05 (1.00-1.10)		1.19 (1.12-1.27)	
Marital status					
Married	10 255	1.04 (0.99-1.10)	.61	1.20 (1.13-1.29)	.11
Single	1346	1.02 (0.90-1.15)		1.07 (0.91-1.27)	
Smoking					
No	6556	1.02 (0.95-1.09)	.56	1.16 (1.07-1.27)	.73
Yes	5045	1.06 (0.98-1.14)		1.23 (1.13-1.34)	
Drinking					
No	6162	1.04 (0.97-1.11)	.83	1.15 (1.06-1.26)	.48
Yes	5439	1.03 (0.96-1.11)		1.23 (1.12-1.34)	
Sleep time					
≤6	5732	1.04 (0.97-1.11)	.92	1.14 (1.05-1.24)	.09
>6	5869	1.03 (0.96-1.11)		1.24 (1.14-1.36)	
Diabetes					
No	10 521	1.04 (0.99-1.10)	.88	1.21 (1.13-1.30)	.07
Yes	1080	1.04 (0.92-1.16)		1.08 (0.93-1.26)	
Heart disease					
No	9674	1.02 (0.96-1.08)	.41	1.20 (1.11-1.29)	.42
Yes	1927	1.07 (0.98-1.17)		1.18 (1.06-1.31)	
Cancer history					
No	11 414	1.04 (0.99-1.09)	.66	1.19 (1.12-1.26)	.99
Yes	187	1.04 (0.66-1.64)		1.08 (0.63-1.85)	

Abbreviation: AAEs, adverse adulthood experiences; ACEs, adverse childhood experiences; CI, confidence interval; HR, hazard ratio.

[a] The analysis was performed in Model 3 (adjusted with age, sex, education, marital status, smoking status, drinking status, sleep, diabetes, diabetes medication, heart disease and cancer history).

[b] P value for interaction was derived by including a multiplicative interaction term in the Cox proportional hazards model. [c] This category includes individuals with no formal education, primary school, or secondary school education levels.

© 2026 Chen B et al. *JAMA Network Open*.

eTable 17. Sensitivity and subgroup analyses of the associations of ACEs and AAEs with incident dementia, stratified by SES, parental education, and residential type

Subgroups	Count	ACEs HR (95% CI) [a]	P interaction [b]	AAEs HR (95% CI) [a]	P interaction [b]
SES					
Low	2089	1.18 (1.05-1.33)		1.25 (1.08-1.45)	
Lower-middle	5551	1.06 (0.97-1.16)	.21	1.22 (1.09-1.37)	.47
Upper-middle	3789	1.11 (0.97-1.26)		1.17 (0.99-1.39)	
High	172	6.12 (0.12-304.32)		NA	
Parental education					
Above high school	254	0.80 (0.19-3.39)	.79	2.24 (0.90-5.57)	.74
Others [c]	11 347	1.11 (1.04-1.18)		1.22 (1.13-1.32)	
Residential type					
Rural	7380	1.13 (1.05-1.21)	.56	1.21 (1.10-1.33)	.40
Urban	4221	1.08 (0.96-1.21)		1.25 (1.09-1.44)	

Abbreviation: AAEs, adverse adulthood experiences; ACEs, adverse childhood experiences; CI, confidence interval; HR, hazard ratio; NA, not applicable; SES, socioeconomic status.

[a] The analysis was adjusted with age, sex, education, marital status, smoking status, drinking status, sleep, diabetes, diabetes medication, heart disease, cancer history, SES, parental education and residential type.

[b] P value for interaction was derived by including a multiplicative interaction term in the Cox proportional hazards model. [c] This category includes individuals with no formal education, primary school, or secondary school education levels.

eTable 18. Sensitivity and subgroup analyses of the associations of ACEs and AAEs with incident stroke, stratified by SES, parental education, and residential type

Subgroups	Count	ACEs HR (95% CI) [a]	P interaction [b]	AAEs HR (95% CI) [a]	P interaction [b]
SES					
Low	2089	1.08 (0.97-1.20)		1.10 (0.97-1.26)	
Lower-middle	5551	1.04 (0.97-1.12)	.35	1.21 (1.11-1.32)	.54
Upper-middle	3789	1.02 (0.92-1.12)		1.21 (1.07-1.36)	
High	172	0.41 (0.20-0.85)		0.63 (0.24-1.65)	
Parental education					
Above high school	254	0.91 (0.62-1.34)	.44	1.18 (0.79-1.76)	.48
Others [c]	11 347	1.04 (0.99-1.10)		1.18 (1.11-1.26)	
Residential type					
Rural	7380	1.08 (1.01-1.14)	.10	1.20 (1.11-1.30)	.59
Urban	4221	0.98 (0.90-1.06)		1.15 (1.04-1.28)	

Abbreviation: AAEs, adverse adulthood experiences; ACEs, adverse childhood experiences; CI, confidence interval; HR, hazard ratio; SES, socioeconomic status.

© 2026 Chen B et al. *JAMA Network Open*.

[a] The analysis was adjusted with age, sex, education, marital status, smoking status, drinking status, sleep, diabetes, diabetes medication, heart disease, cancer history, SES, parental education and residential type.

[b] P value for interaction was derived by including a multiplicative interaction term in the Cox proportional hazards model. [c] This category includes individuals with no formal education, primary school, or secondary school education levels.

© 2026 Chen B et al. *JAMA Network Open*.

eTable 19. Associations of individual components of ACEs and AAEs with incident dementia

	HR (95% CI) [a]	P value
Individual components of ACEs		
Physical abuse	1.11 (0.91-1.36)	.30
Emotional neglect	1.00 (0.83-1.22)	.98
Household substance abuse	1.24 (0.90-1.70)	.19
Household mental illness	1.83 (1.46-2.30)	<.001
Domestic violence	1.36 (1.02-1.81)	.03
Incarcerated household member	2.17 (0.81-5.83)	.12
Parental separation	NA	NA
Unsafe neighborhood	1.17 (0.86-1.59)	.31
Bullying	1.18 (0.91-1.52)	.20
Parental death	1.07 (0.83-1.37)	.61
Sibling death	0.88 (0.70-1.10)	.27
Parental disability	1.31 (1.06-1.61)	.01
Individual components of AAEs		
Death of the child	1.17 (0.92-1.49)	.21
Experiencing lifetime discrimination	1.31 (0.99-1.72)	.06
Ever being confined to bed	1.57 (1.26-1.96)	<.001
Ever being hospitalized	1.75 (1.39-2.21)	<.001
Ever leaving a job	1.51 (1.21-1.87)	<.001

Abbreviation: AAEs, adverse adulthood experiences; ACEs, adverse childhood experiences; CI, confidence interval; HR, hazard ratio; NA, not applicable.

[a] The analysis was performed in Model 3 (adjusted with age, sex, education, marital status, smoking status, drinking status, sleep, diabetes, diabetes medication, heart disease and cancer history).

© 2026 Chen B et al. *JAMA Network Open*.

eTable 20. Associations of individual components of ACEs and AAEs with incident stroke

	HR (95% CI) [a]	P value
Individual components of ACEs		
Physical abuse	1.12 (0.96-1.31)	.15
Emotional neglect	1.04 (0.90-1.21)	.57
Household substance abuse	1.17 (0.91-1.50)	.23
Household mental illness	1.18 (0.96-1.45)	.12
Domestic violence	1.04 (0.81-1.33)	.76
Incarcerated household member	1.57 (0.65-3.79)	.32
Parental separation	NA	NA
Unsafe neighborhood	1.18 (0.93-1.50)	.18
Bullying	1.00 (0.82-1.23)	.98
Parental death	0.95 (0.77-1.17)	.63
Sibling death	0.95 (0.80-1.12)	.52
Parental disability	1.05 (0.89-1.24)	.58
Individual components of AAEs		
Death of the child	1.17 (0.95-1.43)	.13
Experiencing lifetime discrimination	1.16 (0.93-1.45)	.19
Ever being confined to bed	1.45 (1.22-1.72)	<.001
Ever being hospitalized	1.67 (1.39-2.00)	<.001
Ever leaving a job	1.39 (1.18-1.65)	<.001

Abbreviation: AAEs, adverse adulthood experiences; ACEs, adverse childhood experiences; CI, confidence interval; HR, hazard ratio; NA, not applicable.

[a] The analysis was performed in Model 3 (adjusted with age, sex, education, marital status, smoking status, drinking status, sleep, diabetes, diabetes medication, heart disease and cancer history).

© 2026 Chen B et al. *JAMA Network Open*.

eTable 21. Sensitivity analyses for associations of ACEs and AAEs with incident dementia excluded all samples with missing covariates (N = 9360)

	HR (95% CI) [a]	P value
ACEs scores (1-unit per increasing)	1.10 (1.02-1.17)	.01
ACEs category		
0	Reference	NA
1	1.25 (0.91-1.71)	.17
2	1.10 (0.79-1.54)	.56
3	1.40 (0.98-2.00)	.07
≥4	1.55 (1.08-2.24)	.02
P for trend [b]		.02
AAEs scores (1-unit per increasing)	1.21 (1.11-1.32)	<.001
AAEs category		
0	Reference	NA
1	1.61 (1.26-2.06)	<.001
2	1.65 (1.20-2.27)	.002
3	1.82 (1.26-2.64)	.001
≥4	1.81 (1.00-3.27)	.05
P for trend [b]		<.001

Abbreviation: AAEs, adverse adulthood experiences; ACEs, adverse childhood experiences; CI, confidence interval; HR, hazard ratio; NA, not applicable.

[a] The analysis was performed in Model 3 (adjusted with age, sex, education, marital status, smoking status, drinking status, sleep, diabetes, diabetes medication, heart disease and cancer history).

[b] P for trend was calculated by modeling the categorical variable as an ordinal continuous variable in the Cox regression.

© 2026 Chen B et al. *JAMA Network Open*.

eTable 22. Sensitivity analyses for associations of ACEs and AAEs with incident stroke excluded all samples with missing covariates (N = 9360)

	HR (95% CI) [a]	P value
ACEs scores (1-unit per increasing)	1.02 (0.96-1.08)	.55
ACEs category		
0	Reference	NA
1	1.00 (0.80-1.25)	.98
2	0.91 (0.71-1.15)	.43
3	1.07 (0.82-1.40)	.59
≥4	1.10 (0.83-1.46)	.49
P for trend [b]		.50
AAEs scores (1-unit per increasing)	1.18 (1.10-1.26)	<.001
AAEs category		
0	Reference	NA
1	1.09 (0.89-1.34)	.41
2	1.32 (1.03-1.70)	.03
3	1.42 (1.06-1.91)	.02
≥4	2.39 (1.63-3.50)	<.001
P for trend [b]		<.001

Abbreviation: AAEs, adverse adulthood experiences; ACEs, adverse childhood experiences; CI, confidence interval; HR, hazard ratio; NA, not applicable.

[a] The analysis was performed in Model 3 (adjusted with age, sex, education, marital status, smoking status, drinking status, sleep, diabetes, diabetes medication, heart disease and cancer history).

[b] P for trend was calculated by modeling the categorical variable as an ordinal continuous variable in the Cox regression.

© 2026 Chen B et al. *JAMA Network Open*.

eTable 23. Sensitivity analyses for associations of ACEs and AAEs with incident dementia excluded participants with diagnosed as cancer history at baseline (N = 11 414)

	HR (95% CI) [a]	P value
ACEs scores (1-unit per increasing)	1.11 (1.04-1.18)	.002
ACEs category		
0	Reference	NA
1	1.17 (0.87-1.56)	.29
2	1.15 (0.85-1.56)	.36
3	1.35 (0.97-1.88)	.07
≥4	1.58 (1.13-2.21)	.007
P for trend [b]		.006
AAEs scores (1-unit per increasing)	1.24 (1.15-1.34)	<.001
AAEs category		
0	Reference	NA
1	1.54 (1.23-1.94)	<.001
2	1.61 (1.20-2.15)	.001
3	1.74 (1.23-2.47)	.002
≥4	2.62 (1.65-4.16)	<.001
P for trend [b]		<.001

Abbreviation: AAEs, adverse adulthood experiences; ACEs, adverse childhood experiences; CI, confidence interval; HR, hazard ratio; NA, not applicable.

[a] The analysis was adjusted with age, sex, education, marital status, smoking status, drinking status, sleep, diabetes, diabetes medication and heart disease.

[b] P for trend was calculated by modeling the categorical variable as an ordinal continuous variable in the Cox regression.

© 2026 Chen B et al. *JAMA Network Open*.

eTable 24. Sensitivity analyses for associations of ACEs and AAEs with incident stroke excluded participants with diagnosed as cancer history at baseline (N = 11 414)

	HR (95% CI) [a]	P value
ACEs scores (1-unit per increasing)	1.04 (0.99-1.09)	.16
ACEs category		
0	Reference	NA
1	1.04 (0.84-1.28)	.73
2	0.93 (0.75-1.17)	.55
3	1.09 (0.86-1.39)	.48
≥4	1.25 (0.97-1.60)	.09
P for trend [b]		.14
AAEs scores (1-unit per increasing)	1.19 (1.12-1.26)	<.001
AAEs category		
0	Reference	NA
1	1.21 (1.01-1.46)	.04
2	1.40 (1.12-1.76)	.003
3	1.52 (1.16-1.99)	.002
≥4	2.25 (1.58-3.22)	<.001
P for trend [b]		<.001

Abbreviation: AAEs, adverse adulthood experiences; ACEs, adverse childhood experiences; CI, confidence interval; HR, hazard ratio; NA, not applicable.

[a] The analysis was adjusted with age, sex, education, marital status, smoking status, drinking status, sleep, diabetes, diabetes medication and heart disease.

[b] P for trend was calculated by modeling the categorical variable as an ordinal continuous variable in the Cox regression.

© 2026 Chen B et al. *JAMA Network Open*.

eTable 25. Sensitivity analyses for associations of ACEs and AAEs with incident dementia, redefining dementia strictly by cognitive impairment and ADL limitations (N = 11 282)

	**HR (95% CI)** [a]	**P value**
ACEs scores (1-unit per increasing)	1.17 (1.05-1.31)	.005
ACEs category		
0	Reference	NA
1	1.06 (0.64-1.76)	.82
2	0.98 (0.57-1.67)	.93
3	1.06 (0.58-1.95)	.85
≥4	2.04 (1.18-3.53)	.01
P for trend [b]		.004
AAEs scores (1-unit per increasing)	1.37 (1.20-1.56)	<.001
AAEs category		
0	Reference	NA
1	1.69 (1.11-2.58)	.01
2	1.98 (1.20-3.26)	.008
3	2.24 (1.22-4.09)	.009
≥4	3.92 (1.86-8.23)	<.001
P for trend [b]		<.001

Abbreviation: AAEs, adverse adulthood experiences; ACEs, adverse childhood experiences; ADL, Activities of Daily Living; CI, confidence interval; HR, hazard ratio; NA, not applicable.

[a] The analysis was performed in Model 3 (adjusted with age, sex, education, marital status, smoking status, drinking status, sleep, diabetes, diabetes medication, heart disease and cancer history).

[b] P for trend was calculated by modeling the categorical variable as an ordinal continuous variable in the Cox regression.

© 2026 Chen B et al. *JAMA Network Open*.

eTable 26. Sensitivity analyses for associations of ACEs and AAEs with incident dementia using competing risk model

	HR (95% CI) [a]	P value
ACEs scores (1-unit per increasing)	1.12 (1.05-1.19)	<.001
ACEs category		
0	Reference	NA
1	1.19 (0.90-1.59)	.22
2	1.17 (0.87-1.57)	.30
3	1.37 (0.99-1.89)	.06
≥4	1.67 (1.21-2.31)	.002
P for trend [b]		.002
AAEs scores (1-unit per increasing)	1.23 (1.13-1.32)	<.001
AAEs category		
0	Reference	NA
1	1.50 (1.19-1.88)	<.001
2	1.58 (1.18-2.11)	.002
3	1.74 (1.24-2.44)	.001
≥4	2.34 (1.45-3.78)	<.001
P for trend [b]		<.001

Abbreviation: AAEs, adverse adulthood experiences; ACEs, adverse childhood experiences; CI, confidence interval; HR, hazard ratio; NA, not applicable.

[a] The analysis was performed in Model 3 (adjusted with age, sex, education, marital status, smoking status, drinking status, sleep, diabetes, diabetes medication, heart disease and cancer history).

[b] P for trend was calculated by modeling the categorical variable as an ordinal continuous variable in the Cox regression.

© 2026 Chen B et al. *JAMA Network Open*.

eTable 27. Sensitivity analyses for associations of ACEs and AAEs with incident stroke using competing risk model

	HR (95% CI) [a]	P value
ACEs scores (1-unit per increasing)	1.04 (0.99-1.09)	.12
ACEs category		
0	Reference	NA
1	1.05 (0.86-1.29)	.64
2	0.94 (0.76-1.17)	.58
3	1.11 (0.87-1.41)	.41
≥4	1.27 (1.00-1.62)	.05
P for trend [b]		.11
AAEs scores (1-unit per increasing)	1.18 (1.11-1.26)	<.001
AAEs category		
0	Reference	NA
1	1.18 (0.98-1.41)	.08
2	1.37 (1.10-1.71)	.005
3	1.51 (1.16-1.96)	.002
≥4	2.19 (1.54-3.10)	<.001
P for trend [b]		<.001

Abbreviation: AAEs, adverse adulthood experiences; ACEs, adverse childhood experiences; CI, confidence interval; HR, hazard ratio; NA, not applicable.

[a] The analysis was performed in Model 3 (adjusted with age, sex, education, marital status, smoking status, drinking status, sleep, diabetes, diabetes medication, heart disease and cancer history).

[b] P for trend was calculated by modeling the categorical variable as an ordinal continuous variable in the Cox regression.

© 2026 Chen B et al. *JAMA Network Open*.

eTable 28. Sensitivity analyses for associations of ACEs and AAEs with incident dementia additionally adjusted for SES, parental education and residential type

	HR (95% CI) [a]	P value
ACEs scores (1-unit per increasing)	1.11 (1.04-1.18)	.001
ACEs category		
0	Reference	NA
1	1.19 (0.90-1.59)	.22
2	1.16 (0.86-1.57)	.33
3	1.37 (0.99-1.89)	.06
≥4	1.61 (1.16-2.23)	.004
P for trend [b]		.004
AAEs scores (1-unit per increasing)	1.22 (1.13-1.32)	<.001
AAEs category		
0	Reference	NA
1	1.50 (1.19-1.88)	.001
2	1.56 (1.17-2.08)	.002
3	1.73 (1.23-2.43)	.002
≥4	2.31 (1.45-3.68)	<.001
P for trend [b]		<.001

Abbreviation: AAEs, adverse adulthood experiences; ACEs, adverse childhood experiences; CI, confidence interval; HR, hazard ratio; NA, not applicable; SES, socioeconomic status.

[a] The analysis was adjusted with age, sex, education, marital status, smoking status, drinking status, sleep, diabetes, diabetes medication, heart disease, cancer history, SES, parental education and residential type.

[b] P for trend was calculated by modeling the categorical variable as an ordinal continuous variable in the Cox regression.

© 2026 Chen B et al. *JAMA Network Open*.

eTable 29. Sensitivity analyses for associations of ACEs and AAEs with incident stroke additionally adjusted for SES, parental education and residential type

	HR (95% CI) [a]	P value
ACEs scores (1-unit per increasing)	1.04 (0.99-1.09)	.14
ACEs category		
0	Reference	NA
1	1.05 (0.85-1.29)	.65
2	0.93 (0.75-1.16)	.53
3	1.10 (0.86-1.40)	.43
≥4	1.26 (0.98-1.62)	.07
P for trend [b]		.12
AAEs scores (1-unit per increasing)	1.18 (1.11-1.26)	<.001
AAEs category		
0	Reference	NA
1	1.20 (1.00-1.44)	.05
2	1.41 (1.12-1.76)	.003
3	1.51 (1.16-1.97)	.002
≥4	2.21 (1.55-3.14)	<.001
P for trend [b]		<.001

Abbreviation: AAEs, adverse adulthood experiences; ACEs, adverse childhood experiences; CI, confidence interval; HR, hazard ratio; NA, not applicable; SES, socioeconomic status.

[a] The analysis was adjusted with age, sex, education, marital status, smoking status, drinking status, sleep, diabetes, diabetes medication, heart disease, cancer history, SES, parental education and residential type.

[b] P for trend was calculated by modeling the categorical variable as an ordinal continuous variable in the Cox regression.

© 2026 Chen B et al. *JAMA Network Open*.

eTable 30. Sensitivity analyses for associations of ACEs and AAEs with incident dementia additionally adjusted for biomarker (N = 8006)

	HR (95% CI) [a]	P value
ACEs scores (1-unit per increasing)	1.10 (1.02-1.18)	.02
ACEs category		
0	Reference	NA
1	1.18 (0.84-1.65)	.34
2	1.08 (0.76-1.54)	.67
3	1.29 (0.88-1.90)	.20
≥4	1.51 (1.02-2.23)	.04
P for trend [b]		.06
AAEs scores (1-unit per increasing)	1.21 (1.10-1.33)	<.001
AAEs category		
0	Reference	NA
1	1.41 (1.08-1.85)	.01
2	1.37 (0.95-1.96)	.09
3	1.46 (0.94-2.26)	.09
≥4	2.80 (1.68-4.67)	<.001
P for trend [b]		<.001

Abbreviation: AAEs, adverse adulthood experiences; ACEs, adverse childhood experiences; BMI, body mass index; CI, confidence interval; CRP, C-reactive protein; DBP, diastolic blood pressure; HDL, high-density lipoprotein; HR, hazard ratio; NA, not applicable; SBP, systolic blood pressure.

[a] The analysis was adjusted with age, sex, education, marital status, smoking status, drinking status, sleep, diabetes, diabetes medication, heart disease, cancer history, SBP, DBP, BMI, glucose, triglycerides, HDL and CRP.

[b] P for trend was calculated by modeling the categorical variable as an ordinal continuous variable in the Cox regression.

© 2026 Chen B et al. *JAMA Network Open*.

eTable 31. Sensitivity analyses for associations of ACEs and AAEs with incident stroke additionally adjusted for biomarker (N = 8006)

	HR (95% CI) [a]	P value
ACEs scores (1-unit per increasing)	1.03 (0.97-1.09)	.32
ACEs category		
0	Reference	NA
1	0.97 (0.76-1.23)	.78
2	0.93 (0.72-1.21)	.60
3	1.06 (0.80-1.42)	.68
≥4	1.16 (0.86-1.57)	.33
P for trend [b]		.31
AAEs scores (1-unit per increasing)	1.21 (1.13-1.31)	<.001
AAEs category		
0	Reference	NA
1	1.24 (1.00-1.54)	.05
2	1.42 (1.09-1.87)	.01
3	1.52 (1.10-2.12)	.01
≥4	2.72 (1.83-4.05)	<.001
P for trend [b]		<.001

Abbreviation: AAEs, adverse adulthood experiences; ACEs, adverse childhood experiences; BMI, body mass index; CI, confidence interval; CRP, C-reactive protein; DBP, diastolic blood pressure; HDL, high-density lipoprotein; HR, hazard ratio; NA, not applicable; SBP, systolic blood pressure.

[a] The analysis was adjusted with age, sex, education, marital status, smoking status, drinking status, sleep, diabetes, diabetes medication, heart disease, cancer history, SBP, DBP, BMI, glucose, triglycerides, HDL and CRP.

[b] P for trend was calculated by modeling the categorical variable as an ordinal continuous variable in the Cox regression.

© 2026 Chen B et al. *JAMA Network Open*.

eTable 32. Sensitivity analyses for associations of ACEs and AAEs with incident dementia additionally including interaction terms between covariates

	HR (95% CI) [a]	P value
ACEs scores (1-unit per increasing)	1.11 (1.05-1.19)	.001
ACEs category		
0	Reference	NA
1	1.18 (0.89-1.57)	.26
2	1.15 (0.86-1.56)	.35
3	1.35 (0.97-1.87)	.07
≥4	1.65 (1.19-2.29)	.003
P for trend [b]		.003
AAEs scores (1-unit per increasing)	1.23 (1.14-1.33)	<.001
AAEs category		
0	Reference	NA
1	1.55 (1.23-1.94)	<.001
2	1.61 (1.21-2.14)	.001
3	1.70 (1.21-2.39)	.002
≥4	2.46 (1.55-3.91)	<.001
P for trend [b]		<.001

Abbreviation: AAEs, adverse adulthood experiences; ACEs, adverse childhood experiences; CI, confidence interval; HR, hazard ratio; NA, not applicable.

[a] The analysis was adjusted for age, sex, education, marital status, smoking status, drinking status, sleep, diabetes, diabetes medication, heart disease, and cancer history, and additionally included interaction terms between the following variables: age × marital status, diabetes medication × diabetes, and age × diabetes medication.

[b] P for trend was calculated by modeling the categorical variable as an ordinal continuous variable in the Cox regression.

© 2026 Chen B et al. *JAMA Network Open*.

eTable 33. Sensitivity analyses for associations of ACEs and AAEs with incident stroke additionally including interaction terms between covariates

	HR (95% CI) [a]	P value
ACEs scores (1-unit per increasing)	1.04 (0.99-1.09)	.17
ACEs category		
0	Reference	NA
1	1.05 (0.85-1.29)	.64
2	0.95 (0.76-1.18)	.63
3	1.09 (0.85-1.39)	.50
≥4	1.24 (0.97-1.60)	.09
P for trend [b]		.15
AAEs scores (1-unit per increasing)	1.19 (1.12-1.26)	<.001
AAEs category		
0	Reference	NA
1	1.22 (1.02-1.46)	.03
2	1.41 (1.13-1.76)	.002
3	1.50 (1.15-1.95)	.003
≥4	2.35 (1.65-3.34)	<.001
P for trend [b]		<.001

Abbreviation: AAEs, adverse adulthood experiences; ACEs, adverse childhood experiences; CI, confidence interval; HR, hazard ratio; NA, not applicable.

[a] The analysis was adjusted for age, sex, education, marital status, smoking status, drinking status, sleep, diabetes, diabetes medication, heart disease, and cancer history, and additionally included interaction terms between the following variables: age × marital status, sex × heart disease, age × sex, age × diabetes, age × smoking status, smoking status × sleep, smoking status × diabetes medication, diabetes medication × diabetes, age × heart disease, drinking × sleep, sex × sleep, sex × diabetes medication, diabetes × heart disease, and age × diabetes medication.

[b] P for trend was calculated by modeling the categorical variable as an ordinal continuous variable in the Cox regression.

© 2026 Chen B et al. *JAMA Network Open*.

eReferences:

1. Lin L, Wang HH, Lu C, Chen W, Guo VY. Adverse Childhood Experiences and Subsequent Chronic Diseases Among Middle-aged or Older Adults in China and Associations With Demographic and Socioeconomic Characteristics. *JAMA Network Open*. 2021;4(10):e2130143–e2130143. doi:10.1001/jamanetworkopen.2021.30143
2. Wang W, Liu Y, Yang Y, et al. Adverse childhood and adulthood experiences and risk of new-onset cardiovascular disease with consideration of social support: a prospective cohort study. *BMC Medicine*. 2023/08/08 2023;21(1):297. doi:10.1186/s12916-023-03015-1
3. Zhang Y-B, Chen C, Pan X-F, et al. Associations of healthy lifestyle and socioeconomic status with mortality and incident cardiovascular disease: two prospective cohort studies. *BMJ*. 2021;373:n604. doi:10.1136/bmj.n604
4. Liu Y, Wu Y, Cai J, et al. Is there a common latent cognitive construct for dementia estimation across two Chinese cohorts? *Alzheimer's & Dementia: Diagnosis, Assessment & Disease Monitoring*. 2022;14(1):e12356. doi:https://doi.org/10.1002/dad2.12356
5. Zeng M, Chen Y, Lobanov-Rostovsky S, et al. Adiposity and dementia among Chinese adults: longitudinal study in the China Health and Retirement Longitudinal Study (CHARLS). *International Journal of Obesity*. 2025/04/01 2025;49(4):706–714. doi:10.1038/s41366-024-01698-x
6. Peng L, Xiang Q, Jia G, Yin R. Association between sarcopenic obesity and dementia in the Chinese elderly using different definitions of obesity: evidence from the CHARLS. Original Research. *Frontiers in Aging Neuroscience*. 2025–June–04 2025;Volume 17 - 2025doi:10.3389/fnagi.2025.1540272
7. Liu Y, Gao X, Zhang Y, et al. Geographical variation in dementia prevalence across China: a geospatial analysis. *Lancet Reg Health West Pac*. Jun 2024;47:101117. doi:10.1016/j.lanwpc.2024.101117
8. Li C, Luo C, Zhu J, et al. The impact of ADL disability in middle-aged and older adults on the incidence of hip fractures and the mediating role of depression: a longitudinal evidence from CHARLS. *Front Med (Lausanne)*. 2025;12:1604729. doi:10.3389/fmed.2025.1604729
9. Zhang L, Ding H, Liu F, et al. Associations of handgrip strength, walking times, and dementia among Chinese adults: A cross-sectional analysis from CHARLS. *Journal of Affective Disorders*. 2025/12/15/ 2025;391:120028. doi:https://doi.org/10.1016/j.jad.2025.120028
10. Zhao Y, Hu Y, Smith JP, Strauss J, Yang G. Cohort profile: the China Health and Retirement Longitudinal Study (CHARLS). *Int J Epidemiol*. Feb 2014;43(1):61–8. doi:10.1093/ije/dys203
11. Chen S, Chen X, Hou X, Fang H, Liu GG, Yan LL. Temporal trends and disparities of population attributable fractions of modifiable risk factors for dementia in China: a time-series study of the China health and retirement longitudinal study (2011-2018). *Lancet Reg Health West Pac*. Jun 2024;47:101106. doi:10.1016/j.lanwpc.2024.101106
12. Wang J, Jiao D, Zhao X, et al. Childhood Loneliness and Cognitive Decline and

Dementia Risk in Middle-Aged and Older Adults. *JAMA Network Open*. 2025;8(9):e2531493–e2531493. doi:10.1001/jamanetworkopen.2025.31493
13. Mohebbi M, Nguyen V, McNeil JJ, et al. Psychometric properties of a short form of the Center for Epidemiologic Studies Depression (CES-D-10) scale for screening depressive symptoms in healthy community dwelling older adults. *Gen Hosp Psychiatry*. Mar–Apr 2018;51:118–125. doi:10.1016/j.genhosppsych.2017.08.002
14. Zuo X, Chen Y. Association between activities of daily living and depression symptoms among older adults in China: A nationally representative cross-sectional survey. *BMC Psychology*. 2025/08/30 2025;13(1):989. doi:10.1186/s40359-025-03223-9
15. Maj M, Stein DJ, Parker G, et al. The clinical characterization of the adult patient with depression aimed at personalization of management. *World Psychiatry*. Oct 2020;19(3):269–293. doi:10.1002/wps.20771
16. Zhu J, Chiu MM. Gender- and age-bias in CES-D when measuring depression in China: A Rasch analysis. *Current Psychology*. 2023/04/01 2023;42(10):8186–8196. doi:10.1007/s12144-021-01991-2
17. Zhou Y, Kivimäki M, Yan LL, et al. Associations between socioeconomic inequalities and progression to psychological and cognitive multimorbidities after onset of a physical condition: a multicohort study. *eClinicalMedicine*. 2024;74doi:10.1016/j.eclinm.2024.102739
18. Luo S, Chen W, Hu W, et al. Parental Education, Own Education, and Cognitive Function in Middle-Aged and Older Adults. *JAMA Network Open*. 2025;8(5):e2513036–e2513036. doi:10.1001/jamanetworkopen.2025.13036
