# Supplementary material for: Life-Course Psychosocial Stress and Risk of Dementia and Stroke in Middle-Aged and Older Adults
Source: JAMA Netw Open. 2026 Jan 28;9(1):e2556012. doi: 10.1001/jamanetworkopen.2025.56012 (PMC12853204; doi:10.1001/jamanetworkopen.2025.56012)
Supplement: Supplement 2. — Data Sharing Statement [file jamanetwopen-e2556012-s002.pdf]

Data Sharing Statement

Chen. Life-Course Psychosocial Stress and Risk of Dementia and Stroke in Middle-Aged and Older Adults. *JAMA Netw Open*. Published January 28, 2026.
doi:10.1001/jamanetworkopen.2025.56012

Data
Data available: Yes
Data types: Other (please specify)
Additional Information: Yes
How to access data: The CHARLS datasets were available at http://charls.pku.edu.cn/en/.
When available: With publication

Supporting Documents
Document types: None

Additional Information
Who can access the data: Data from the China Health and Retirement Longitudinal Study (CHARLS) are available to all researchers upon making an application.
Types of analyses: All types of analyses with an application approved.
Mechanisms of data availability: After approval of an application.
